# Supplementary material for: High-entropy alloys catalyzing polymeric transformation of water pollutants with remarkably improved electron utilization efficiency
Source: Nat Commun. 2025 Jan 2;16:148. doi: 10.1038/s41467-024-55627-7 (PMC11697309; doi:10.1038/s41467-024-55627-7)
Supplement: Supplementary file 1 — Supplementary Information [file 41467_2024_55627_MOESM1_ESM.pdf]

Supplementary Information for

High-entropy alloys catalyzing polymeric transformation of water pollutants with remarkably improved electron utilization efficiency

Ziwei Yao[1], Yidi Chen[1]*, Xiaodan Wang[1], Kusheng Hu[2], Shiying Ren[2], Jinqiang Zhang[2], Zhao Song[3], Nanqi Ren[1], Xiaoguang Duan[2]*

[1] State Key Laboratory of Urban Water Resource and Environment, Shenzhen Key Laboratory of Organic Pollution Prevention and Control, School of Civil and Environmental Engineering, Harbin Institute of Technology, Shenzhen, Guangdong 518055, P. R. China

[2] School of Chemical Engineering, The University of Adelaide, Adelaide, SA 5005, Australia

[3] School of Materials and Environmental Engineering, Shenzhen Polytechnic University, Shenzhen 518055, P. R. China

Yidi Chen*, Xiaoguang Duan*

*To whom correspondence may be addressed.

Email: chenyidi@hit.edu.cn, xiaoguang.duan@ adelaide.edu.au

This PDF file includes:

Supplementary Methods
Supplementary Figure 1 to 51
Supplementary Table 1 to 7
Supplementary References

Supplementary Methods

Reagents and materials. Palladium (II) acetylacetonate ($Pd(acac)_2$, 99%), Cupric acetylacetonate ($Cu(acac)_2$, 97%), Cobalt(III) acetylacetonate ($Co(acac)_3$, 98%), Iron(III) acetylacetonate ($Fe(acac)_3$), 2,4,6-tri-tertbutylphenol (BHT), and 9,10-Diphenylanthracene (DPA, 98%) were purchased from Aladdin. Oleylamine (OAm, >70%) and OXONE® monopersulfate compound (PMS) were bought from Sigma-Aldrich. Potassium hydroxide (KOH, 99.95%) and Ethanol (99.5%) were purchased from Macklin. nickel (II) acetylacetonate ($Ni(acac)_2$, 98%), glucose (98%), N,N,N-Trimethylhexadecan-1-aminium chloride (CTAC, 96%), Urea(CH_4N_2O, 99%), 2,2,6,6-Tetramethylpiperidine (TEMP, 98%), 5,5-Dimethyl-1-pyrroline N-oxide (DMPO, 97%) were supplied by Bidepharm (Shanghai, China). Other reagents not mentioned were purchased from Aladdin Chemical Regent Company. The actual water bodies (Tap water, Dasha River) were sampled separately in Shenzhen, Guangdong Province. Except for the actual water matrix test, all experiments were conducted using deionized water.

Catalytic Effect Test. Typically, 5 mg catalyst was added into 50 mL phenol solution. Subsequently, KOH and H_2SO_4 were added to adjust pH if necessary, and various inorganic salts were also added at this stage if required. The corresponding concentration of PMS was added at the beginning of the experiment. After sampling at different time points, the mixture was filtered using a filter head, and the concentration of phenol was detected using UPLC.

ABTS colorimetric method for the measurement of PMS. Diluted sample was added into 2 mL acetate buffered solution (pH 4) containing ABTS (2 mM) and 15 μM iodide (as a catalyst) to react for 20 min and then was diluted to 5 ml. The resulting solution was analyzed by UV−vis spectrometer at the wavelength of 415 nm (ε=34000 M^{-1} cm^{-1}).

***In Situ* Raman spectroscopy.** In situ Raman spectroscopic measurements were carried out on a confocal Raman microscope (alpha300R, WiTech). Initially, 0.5 mg of catalyst was added to a glass groove containing 100 μL of PMS. A semiconductor TEM single-frequency laser with a wavelength of 532 nm was used as the excitation light source, providing a laser energy of 15 mW and utilizing a grating with a density of 600 g mm^{-1}. It was focused by 20 times long focal lens to vertically illuminate the surface of the catalyst and recorded the spectrum in the range of 400 to 2,200 cm^{-1} was recorded with an integration time of 45s for six times. The data were processed by simple cosmic ray subtraction and weighted average method.

Electrochemical Characterization. The electrochemical properties of these filter electrodes were characterized in a three electrode configuration using a CHI 760E electrochemical workstation. The working electrode was prepared through the following procedure: Initially, an electrode solution was prepared by dispersing 3 mg of catalyst in a mixture of 2 mL of ethanol and 100 μL of a 5% Nafion solution (the binder), followed by overnight stirring. The drop-casting method was used to apply 10 μL of electrode ink onto the surface of a polished glassy carbon electrode (GCE). The electrode was then dried at 60°C for 10 minutes. This process was repeated multiple times until the catalyst completely covered the surface of the glassy carbon electrode. All of the current densities reported in this work are based on geometric surface area.

(1) Open circuit potential method (OCPT). Firstly, the prepared HEAs-GCE and NC-GCE are soaked in the reaction solution (20 mM Na_2SO_4) overnight to maintain a stable potential. Then the open circuit potential of HEAs-GCE or NC-GCE was monitored by open circuit potential analysis using a saturated silver chloride electrode as the reference electrode, and all the potential values were normalized to the reversible hydrogen electrode (RHE) before testing. After stabilizing the open circuit potential of system, PMS and phenol were sequentially added to the solution to monitor the resulting changes in potential. The experiment was conducted for a duration of 8000 seconds with a sampling interval of 0.1 seconds.

(2) Cyclic voltammetry (CV). CV was performed between -0.25 V and 1.1 V at a scan rate of 10 mV s^{-1} using an electrochemical workstation. The experiments were conducted in phenol solution, prepared by 50mM Na_2SO_4 solution, with a three-electrodes-cell configuration including a working electrode (10×10×1 mm graphite plate electrode), a counter electrode (platinum electrode), and a reference electrode (saturated silver chloride electrode), and all the potential values were normalized to the reversible hydrogen electrode (RHE) before testing.

(3) Linear sweep voltammetry (LSV). LSV was uses a standard three-electrode configuration, in which the opposite electrode is used as platinum electrode Ag/AgCl/KCl as reference electrode, and graphite plate as the working electrode. The reaction electrolyte was 50 mM Na_2SO_4 solution and phenol solution with a pH of 4. The electrochemical parameters tested were initial potential (−0.25 V), final potential (1.1 V) and sweep speed (10 mV s^{-1}). The data were fitted by tafel equation to obtain the tafel slopes for comparison of electron transfer rate.

(4) Electrochemical impedance spectroscopy (EIS). To verify the difference in internal resistance of catalysts, EIS was conducted with an alternating current over 10^6–10^{-2} Hz at a 5 mV increment.

(5) Electrochemical active surface area (ECSA). To measure the ECSA of the catalyst, the response current values (I_a and I_c) and double-layer capacitance (C_{dl}) were determined by CV testing at various scan rates. The ECSA can be estimated based on the C_{dl} of the catalytic surface, allowing for a comparison of the activity electrochemical strength between HEAs and NC. CV was performed between 0.2 V and 0.4 V at a scan rate of 30 mV s^{-1}, 40 mV s^{-1}, 60 mV s^{-1}, 80 mV s^{-1}, 100 mV s^{-1} and 150 mV s^{-1}. Other parameters in the experimental system are the same as those in the CV experiment.

(6) Galvanic cell oxidation test. The catalyst was sprayed onto graphite plates to create cathode and anode electrodes. the galvanic experiment took place in an H-type double-chamber electrolytic cell. The cathode chamber was supplied with PMS, and the anode chamber was supplemented with

phenol. Both chambers were separated by a cation exchange membrane, with the anode and cathode connected through an ammeter.

Cell Culture and Cell Viability Assay. The 16HBE cell lines were cultivated in RPMI-1640 medium, a formulation developed by the Roswell Park Memorial Institute, supplemented with 10% fetal bovine serum (FBS) and 1% penicillin/streptomycin (P/S). This carefully balanced composition aims to foster robust cell growth while ensuring the maintenance of a sterile culture environment. The cells were maintained at 37 °C in a humidified incubator with 95% air and 5% CO_2. Cells (2×104 cells/well) were seeded into a 96-well plate and cultured until reaching the appropriate growth status and then treated with various concentrations of tested samples. The concentrations were 0, 25, 50,100, and 200 ppm, respectively, for HEAs. The concentrations were 0, 2.5, 5, 10, and 20 ppm, respectively, for Co^{2+}. The concentrations were 0, 1.25, 2.5, 5, and 10 ppm, respectively, for mixed ions. After a 48-hour incubation period, carefully remove the culture plate. Subsequently, introduce 100 μL of CellTiter-Lumi™ Luminescent Cell Viability Assay Kit (CTL) into each well. Gently shake the plate for 2 minutes to ensure proper mixing, followed by an incubation period of 10 minutes. Remove 70 μL of the reaction mixture from each well and transfer it to a 96-well cell culture plate. Utilize a microplate reader equipped with a luminometer to quantify the bioluminescent signal intensity, facilitating precise assessment of cell viability. All the experiments were performed in three replicates.

Long-term continuous flow Treatment using HEAs. A long-term degradation of phenol test was conducted to evaluate the practical application performance of the HEAs. Fill a custom-designed fixed-bed column (approximately 10 mm in diameter) with 150 mg of HEAs powder and a bed depth of 14 mm. In order to avoid the escape of the HEAs powder from the fixed-bed column during the continuous-flow operation, custom-made polyethylene sieve plates (with a diameter of 10.1 mm, a height of 1.6 mm, and an aperture of 20 μm) were used to fill and compact HEAs in

both the upper and lower layers. Furthermore, polyester fibers were employed for secondary containment and to stabilize the water flow. The experimental solution was prepared using tap water spiked with phenol to simulate wastewater. A phenol feed solution (0.1 mM) and PMS (0.25 mM) were passed through the flow-through reactor at a 1.08 mL min^{-1} flow rate. When the effluent reached 50 mL, the concentration of phenol in the effluent was measured using UPLC to assess the treatment performance of the system.

Measurement of oxidation products. In the reaction product analysis experiments of phenol, the initial concentration of phenol and PMS was selected as 0.1 mM and 0.25 mM, respectively, and the addition amount of catalysts was 0.1 g L^{-1}. Besides, in order to study the products accumulated in solid phases, solid samples were separated by filtration and eluted with organic solvents (tetrahydrofuran and toluene) to extract the transformation products in solid phases. The liquid sample was concentrated through a C18 extraction column and eluted with an organic solvent (methanol) to concentrate the conversion product. The oxidation products of phenol were characterized by ultra-high performance liquid chromatography with quadrupole time of flight initial mass spectrometry (UPLC-QTOF-MS). The HPLC operating conditions were: ZORBAX Eclipse Plus C18 column (2.1×100 mm 1.8-μm), water (mobile phase A), methanol (mobile phase B), add 5mM ammonium acetate to phases A and B, flow rate: 0.3 mL min^{-1}, wavelength: 280 nm, gradient elution program: A:B = 95%:5% (0 min), 95%:5% (1min), 5%:95% (1-6 min), 5%:95% (6-8 min), and 95%:5% (8-10 min), 1.5 μL injection volume, 200 μLcolumn temperature chamber, 40 ℃ column temperature. Mass spectrometry operating conditions: desolventizing gas was nitrogen at a flow rate of 600 L h^{-1}, desolventizing gas temperature was 450 °C; ion source was ESI in negative ion mode; temperature was 100 °C and the collision voltage was set to 2.5 kV.

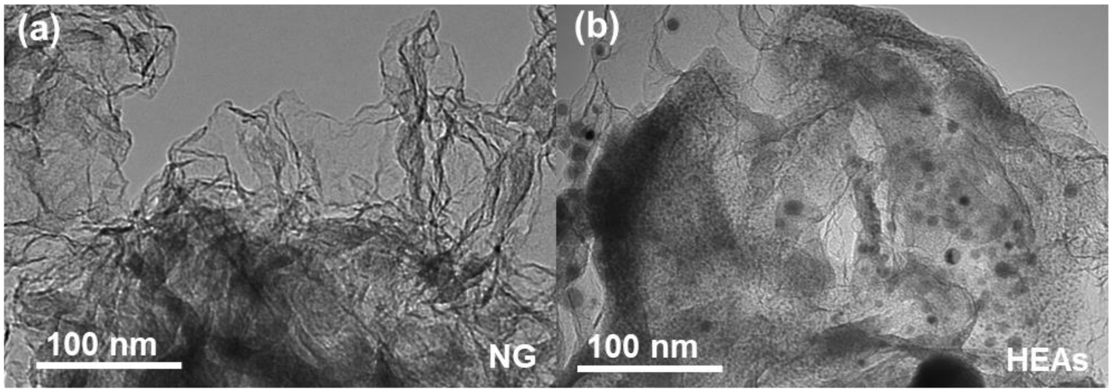

Supplementary Fig.1 TEM images of NG and HEAs.

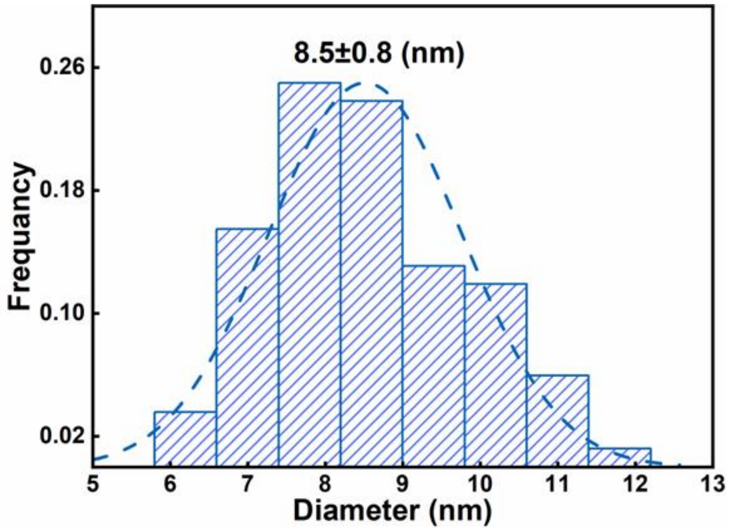

Supplementary Fig.2 The size distribution of $Cu_{12}Pd_{11}Fe_{10}Co_{11}Ni_{12}$ NPs.

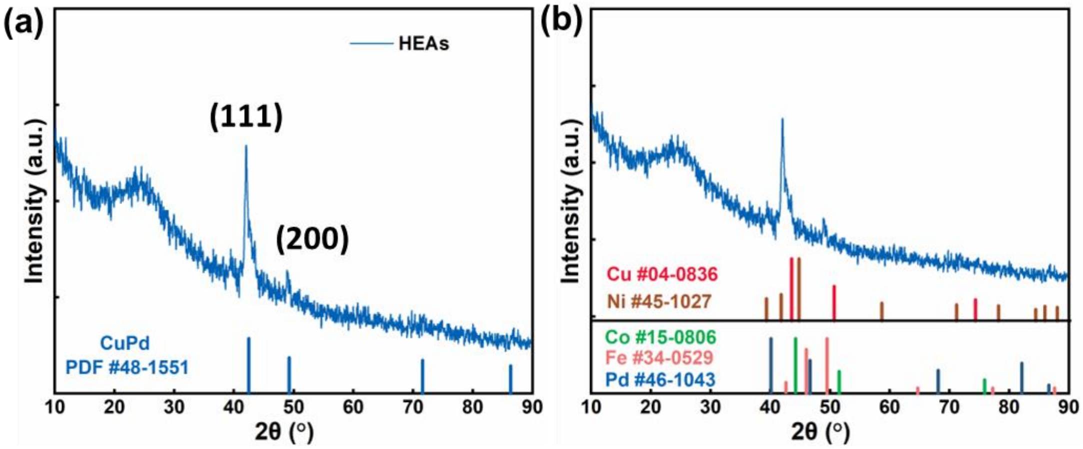

Supplementary Fig.3 XRD pattern HEAs and corresponding standard PDF cards.

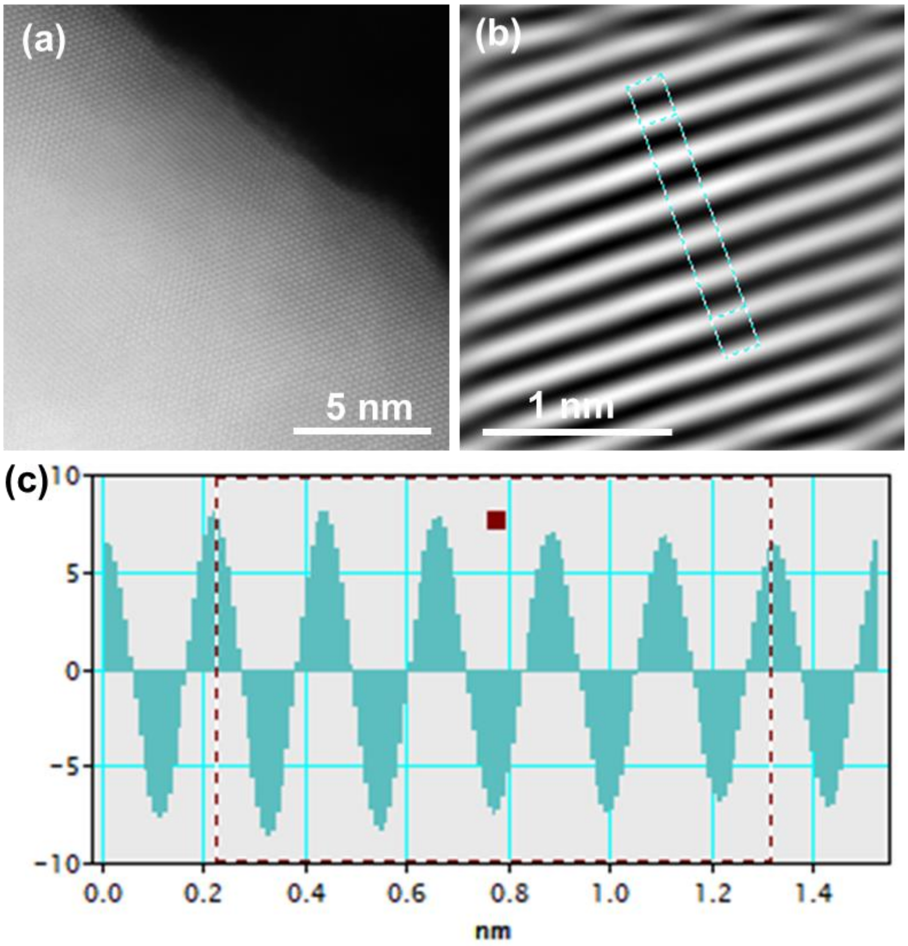

Supplementary Fig.4 (a) Aberration-corrected HAADF-STEM image (scale bar, 5 nm). (b) the corresponding inverse FFT lattice fringes pattern of HEAs and the corresponding interplanar spacing diagram (c).

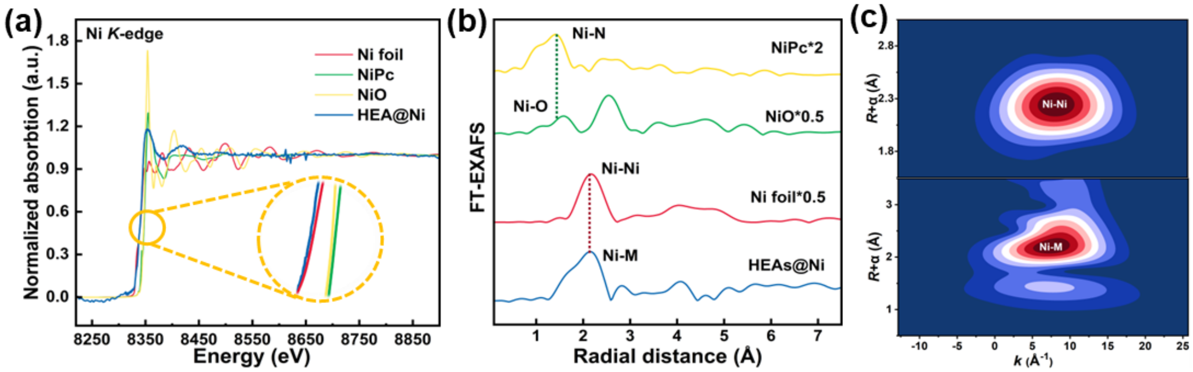

Supplementary Fig.5 (a) XANES at Ni L-edge. (b) FT-EXAFS. (c) WTs of EXAFS spectra of HEAs, NiO, Ni foil, and NiPc.

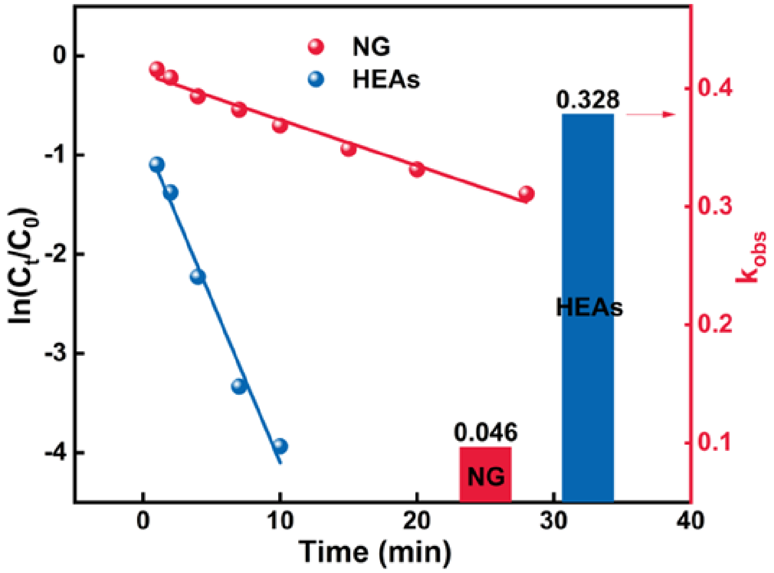

Supplementary Fig.6 Comparison between the apparent rate constants of HEAs and NG. Dosage: $[Phenol]_0$: 0.1 mM, PMS: 0.25 mM, reaction solution: 50 mL, catalyst: 0.1 g L^{-1}.

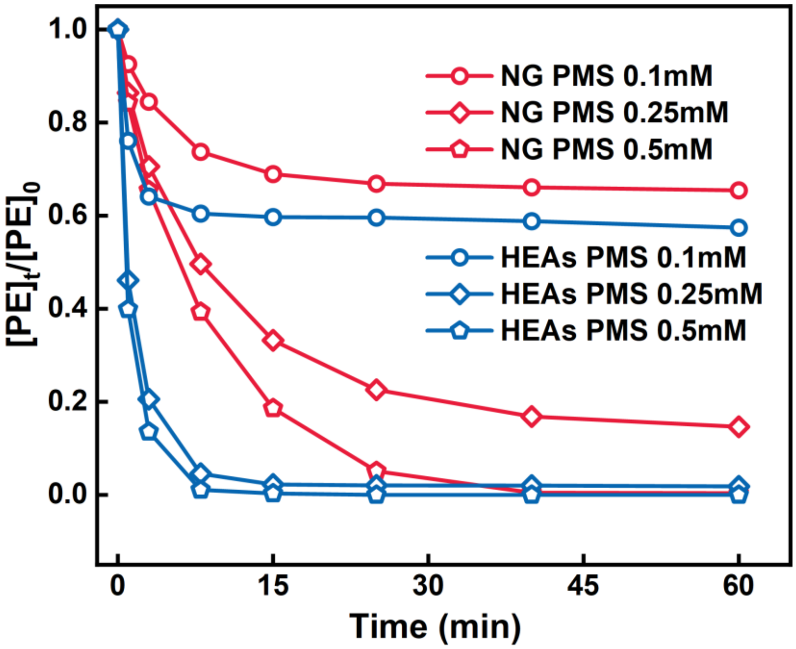

Supplementary Fig.7 Degradation activity of HEAs and NG under different PMS concentrations. Dosage: $[Phenol]_0$: 0.1 mM, reaction solution: 50 mL, catalyst: 0.1 g L^{-1}, reaction time: 60 min.

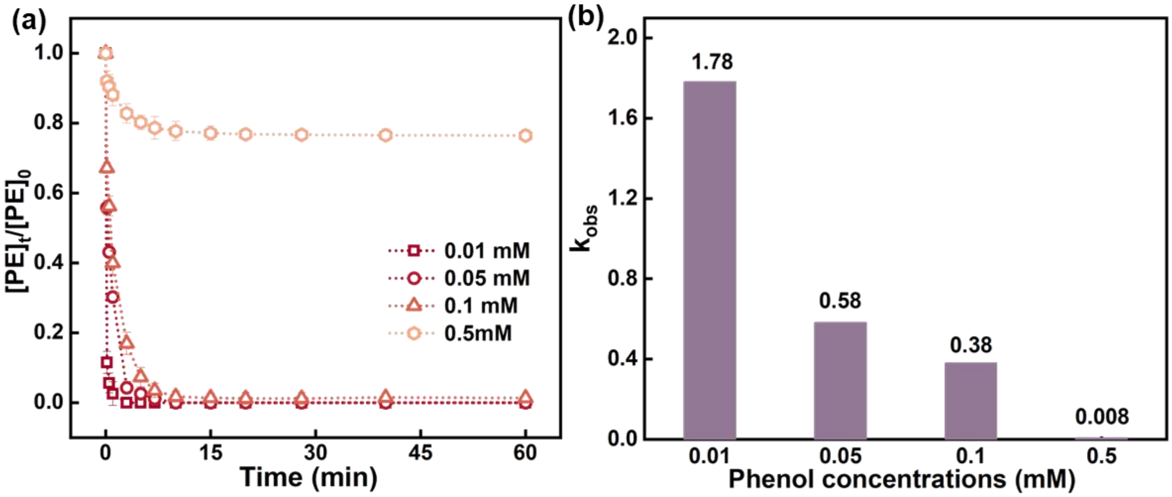

Supplementary Fig.8 The phenol removal performance of different initial phenol concentrations. Error bars represent the standard deviation, obtained by repeating the experiment three times. Conditions: [catalyst] = 0.1 g L^{-1}, reaction solution: 50 mL, [PMS] = 0.25 mM, reaction time: 60 min..

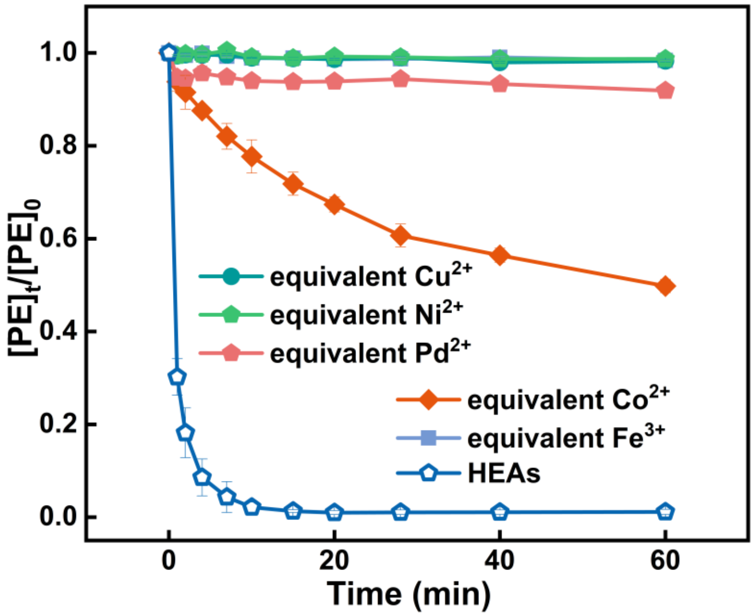

Supplementary Fig.9 Degradation activity of equivalents of ions (quantified by loading on HEA surfaces) is individually employed as catalysts. Error bars represent the standard deviation, obtained by repeating the experiment three times. Dosage: $[Phenol]_0$: 0.1 mM, PMS: 0.25 mM, reaction solution: 50 mL, catalyst: 0.1 g L^{-1}, reaction time: 60 min.

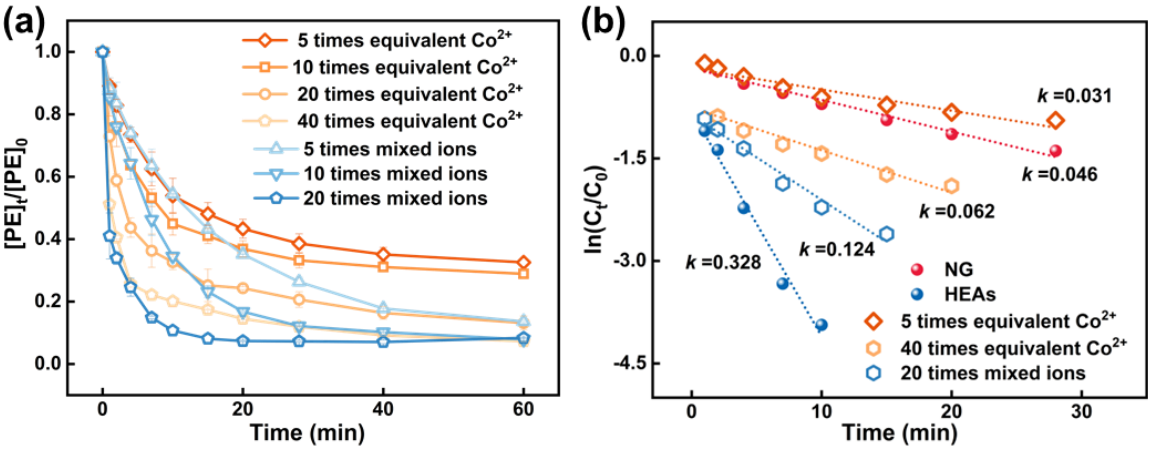

Supplementary Fig.10 (a) Degradation efficiency of phenol when different equivalents of Co^{2+} and mixed ions were used as catalysts. (b) Apparent kinetic constants of NG, HEAs, Co^{2+} and mixed ions. Error bars represent the standard deviation, obtained by repeating the experiment three times. Dosage: PMS: 0.25 mM, reaction solution: 50 mL, $[Pollutants]_0$: 0.1 mM, reaction time: 60 min.

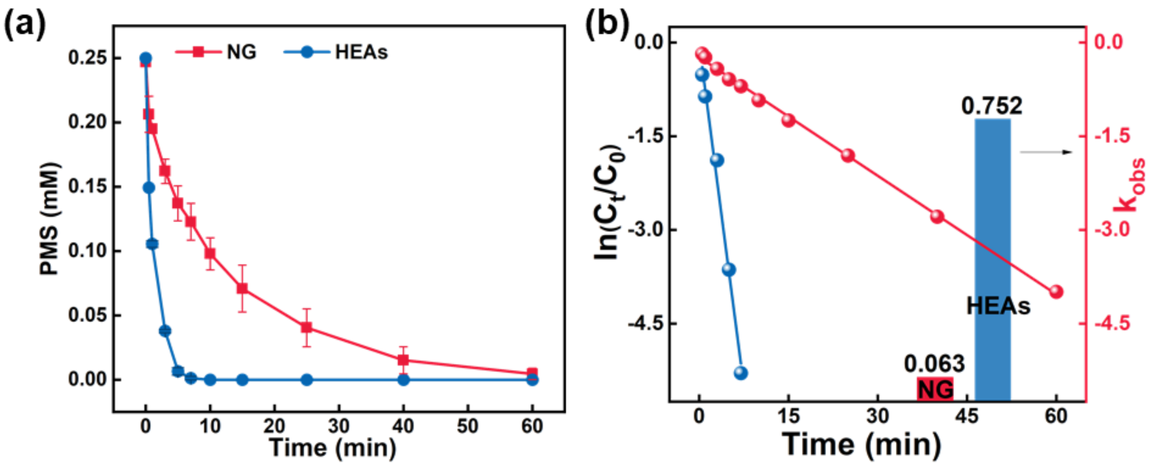

Supplementary Fig.11 (a) Decomposition rate and (b) kinetic constants of PMS with the catalytic effect of HEAs and NG. Error bars represent the standard deviation, obtained by repeating the experiment three times. Dosage: $[Phenol]_0$: 0.1 mM, PMS: 0.25 mM, reaction solution: 50 mL, catalyst: 0.1 g L^{-1}.

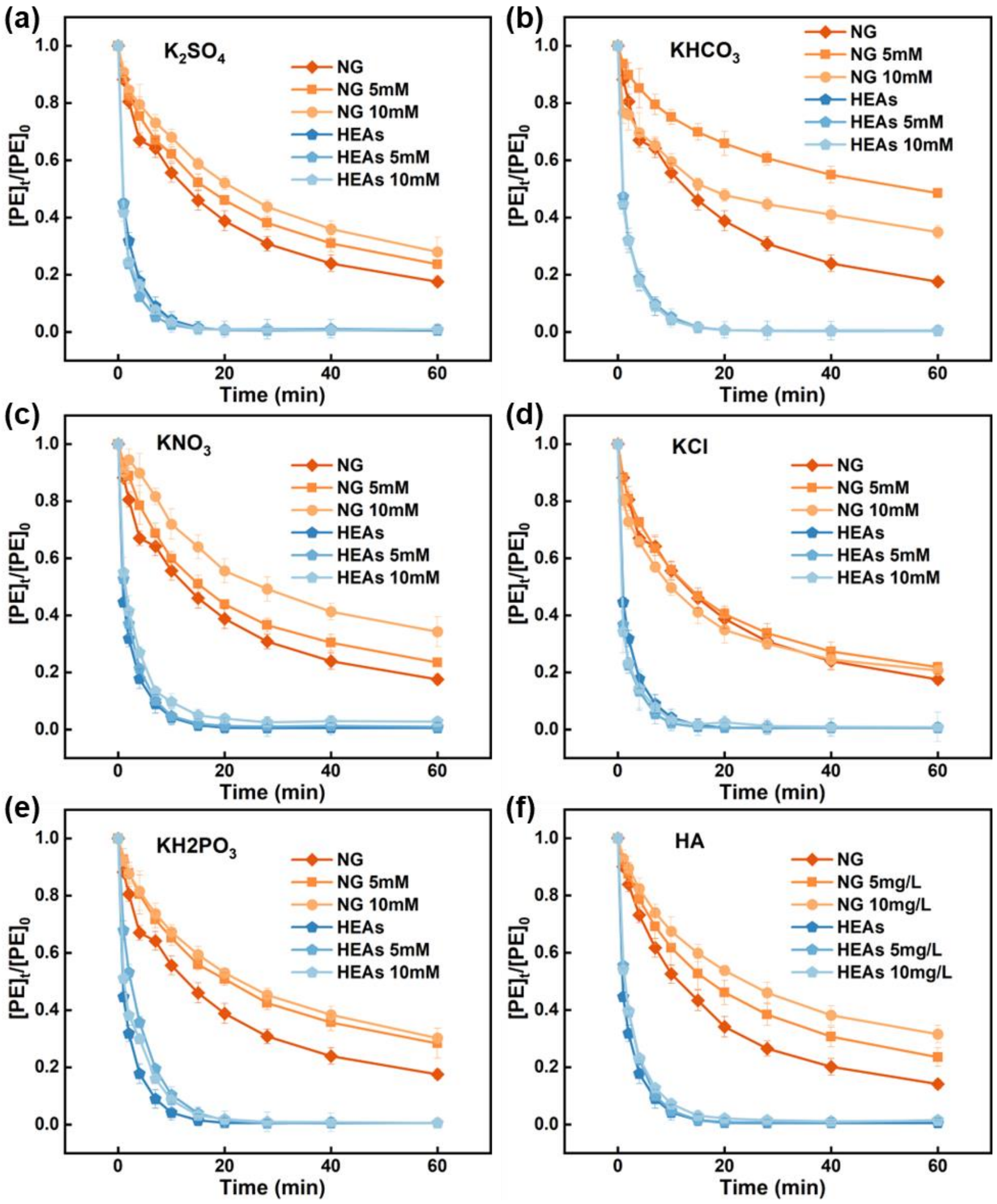

Supplementary Fig.12 HEAs and NG catalytic degradation efficiency of phenol with interference of salts and humic acid. Error bars represent the standard deviation, obtained by repeating the experiment three times. Dosage: $[\text{Phenol}]_0$: 0.1 mM, PMS: 0.25 mM, reaction solution: 50 mL, catalyst: 0.1 g L^{-1}, reaction time: 60 min.

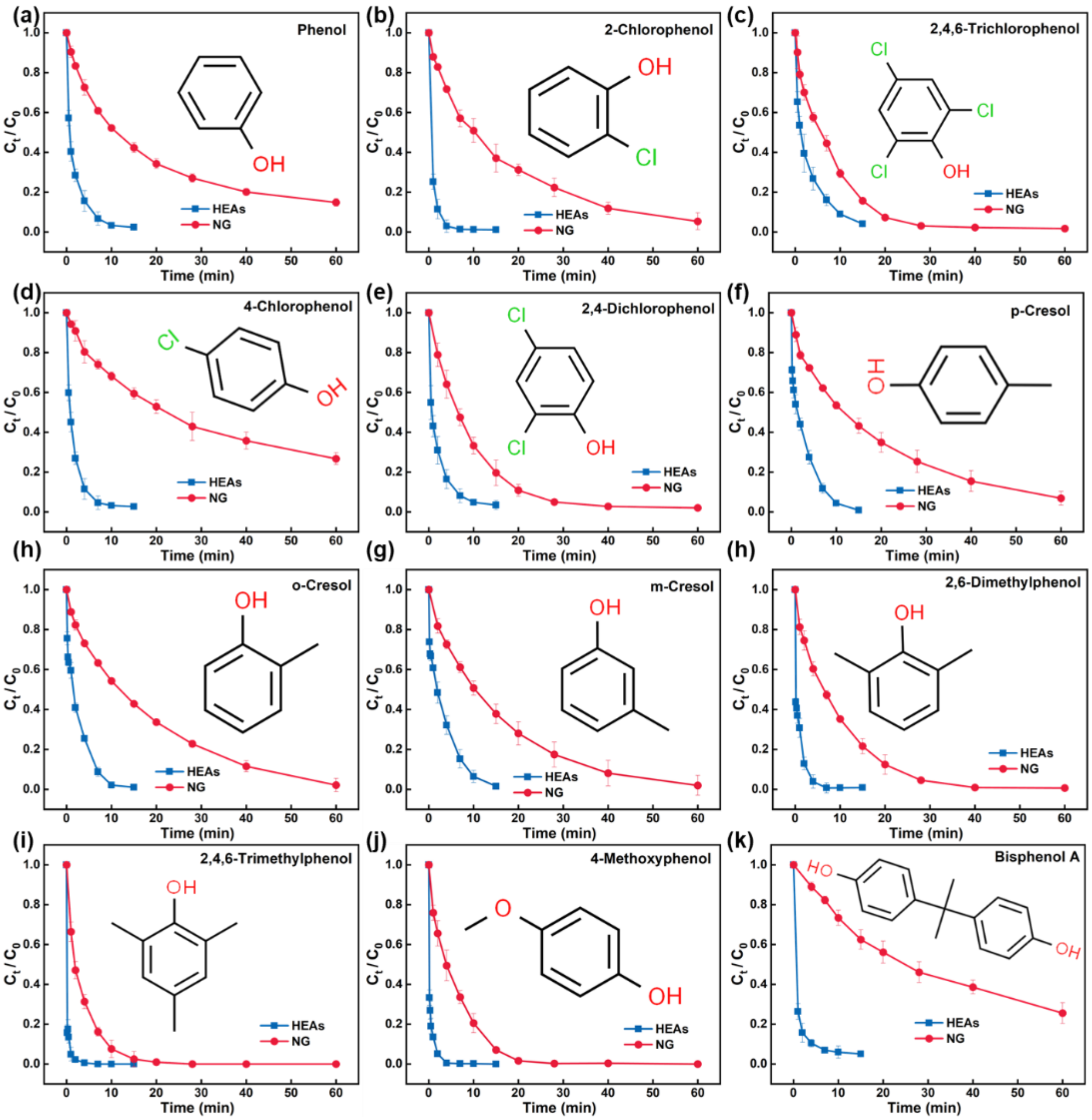

Supplementary Fig.13 Degradation efficiency of different pollutants in the HEAs–PMS system and the NG–PMS system. Error bars represent the standard deviation, obtained by repeating the experiment three times. Dosage: [Pollutants]$_0$: 0.1 mM, PMS: 0.25 mM, reaction solution: 50 mL, catalyst: 0.1 g L^{-1}.

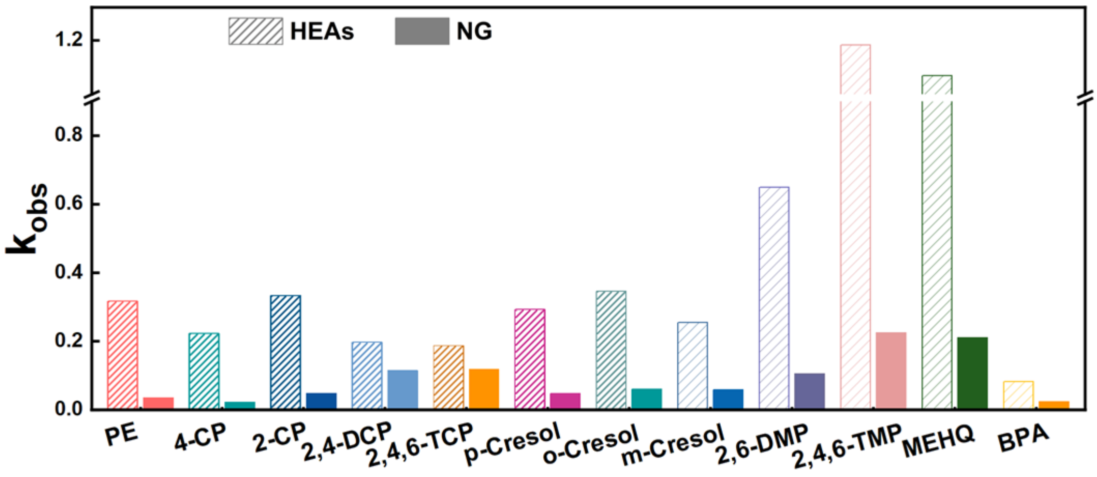

Supplementary Fig.14 Apparent kinetic constants of NG and HEAs degradation different pollutants.

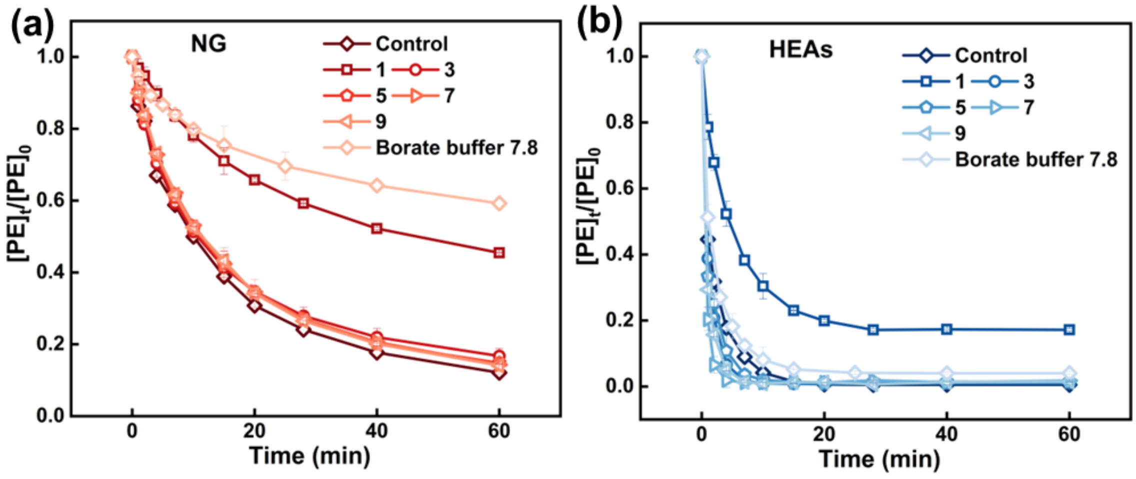

Supplementary Fig.15 Degradation activity of HEAs and NG under different pH and borate buffer environments. Error bars represent the standard deviation, obtained by repeating the experiment three times. Dosage: $[Phenol]_0$: 0.1 mM, PMS: 0.25 mM, reaction solution: 50 mL, catalyst: 0.1 g L^{-1}, reaction time: 60 min.

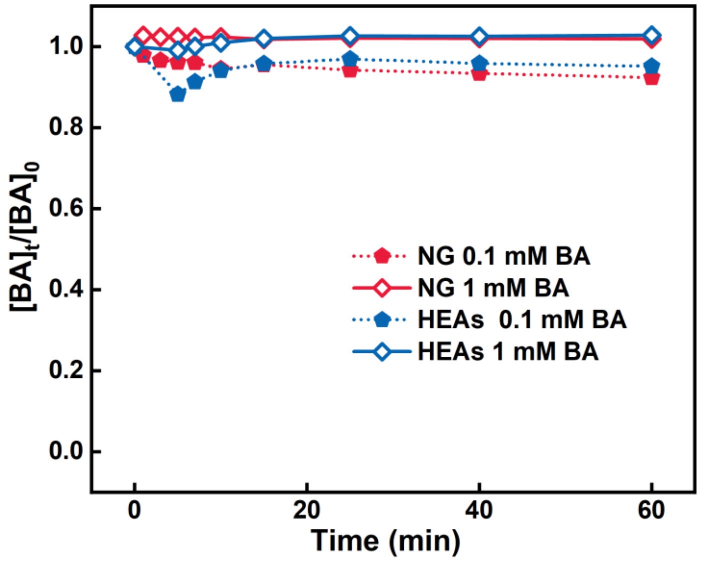

Supplementary Fig.16 Degradation activity of HEAs and NG using BA as probe. Dosage: $[BA]_0$: 0.1 mM/1mM, PMS: 0.25 mM, reaction solution: 50 mL, catalyst: 0.1 g L^{-1}, reaction time: 60 min.

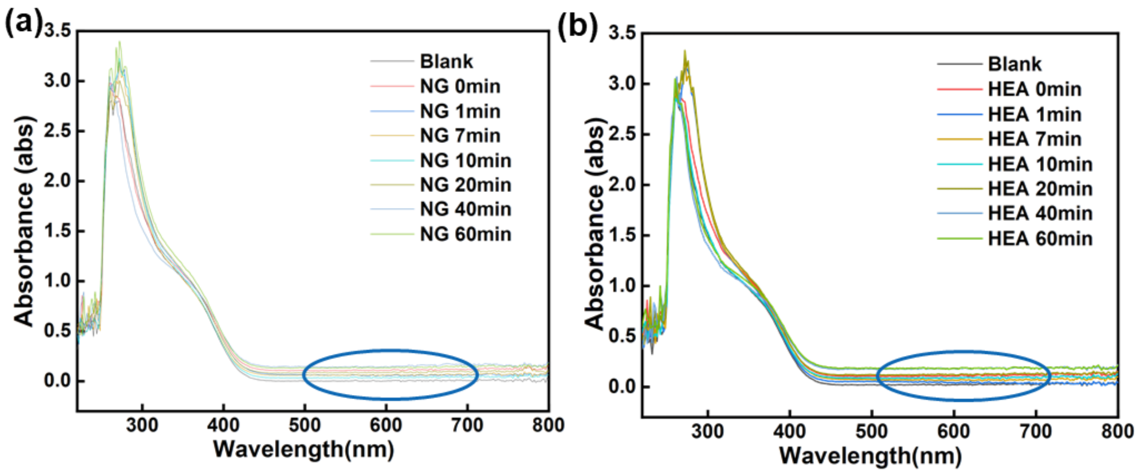

Supplementary Fig.17 The absorption spectra of NBT as a probe in NG-PMS and HEAs-PMS systems. Dosage: $[NBT]_0$: 0.2 mM, PMS: 0.25 mM, reaction solution: 50 mL, catalyst: 0.1 g L^{-1}, reaction time: 60 min.

The absence of monoformazan and diformazan generation within the range of the blue circle is indicated by the lack of absorption peaks between 500 and 700 nm in the UV−vis spectrum.

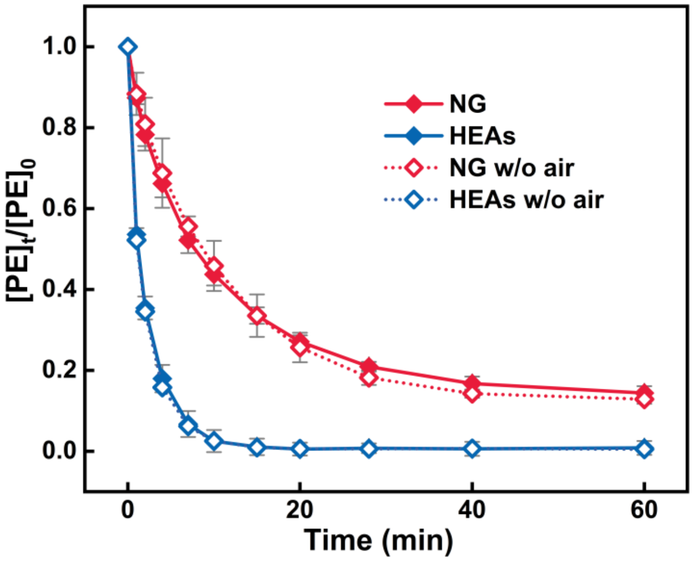

Supplementary Fig.18 Degradation activity of HEAs and NG under air and anaerobic environments. Error bars represent the standard deviation, obtained by repeating the experiment three times. Dosage: $[Phenol]_0$: 0.1 mM, PMS: 0.25 mM, reaction solution: 50 mL, catalyst: 0.1 g L^{-1}, reaction time: 60 min. Anaerobic experiments confirm that oxygen in the air does not participate in the reactions of the HEAs-PMS and NG-PMS systems.

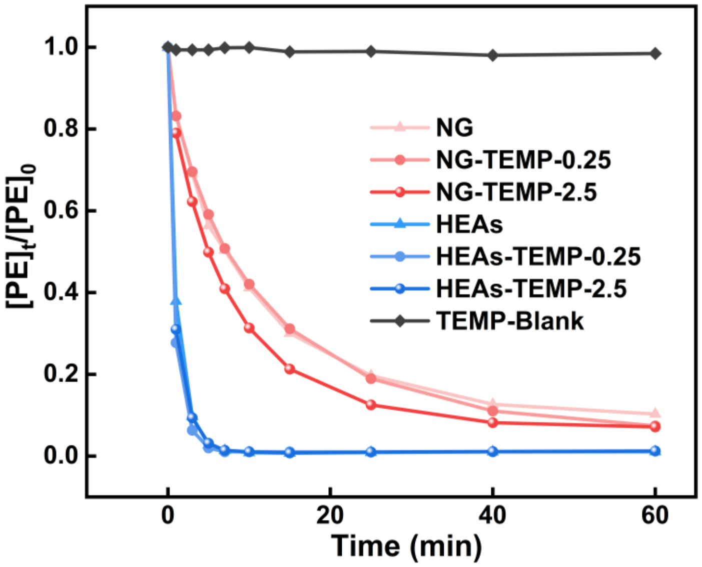

Supplementary Fig.19 Quenching effects by TEMP. Dosage: $[Phenol]_0$: 0.1 mM, PMS: 0.25 mM, $[TEMP]_0$: 0.25/2.5 mM, $[pH]_0$: 4.0, reaction solution: 50 mL, catalyst: 0.1 g L^{-1}.

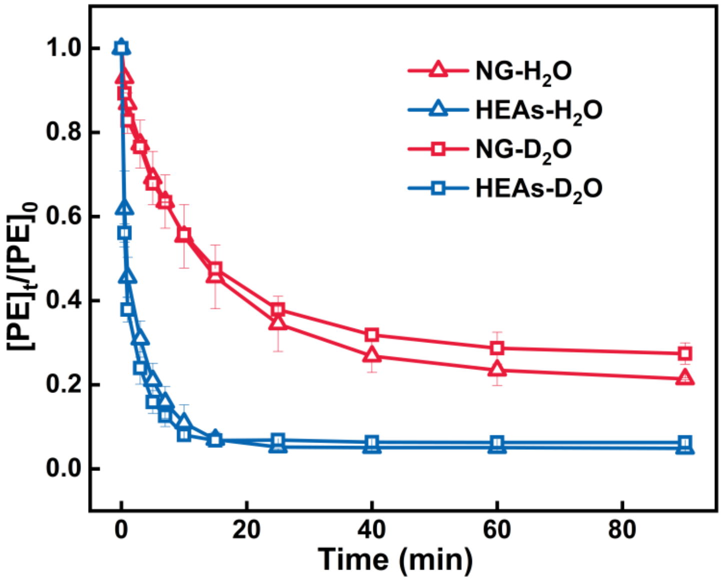

Supplementary Fig.20 Degradation activity of HEAs and NG under H_2O and D_2O environments. Error bars represent the standard deviation, obtained by repeating the experiment three times. Dosage: [Phenol]$_0$: 0.1 mM, PMS: 0.25 mM, reaction solution: 50 mL, catalyst: 0.1 g L^{-1}, reaction time: 90 min.

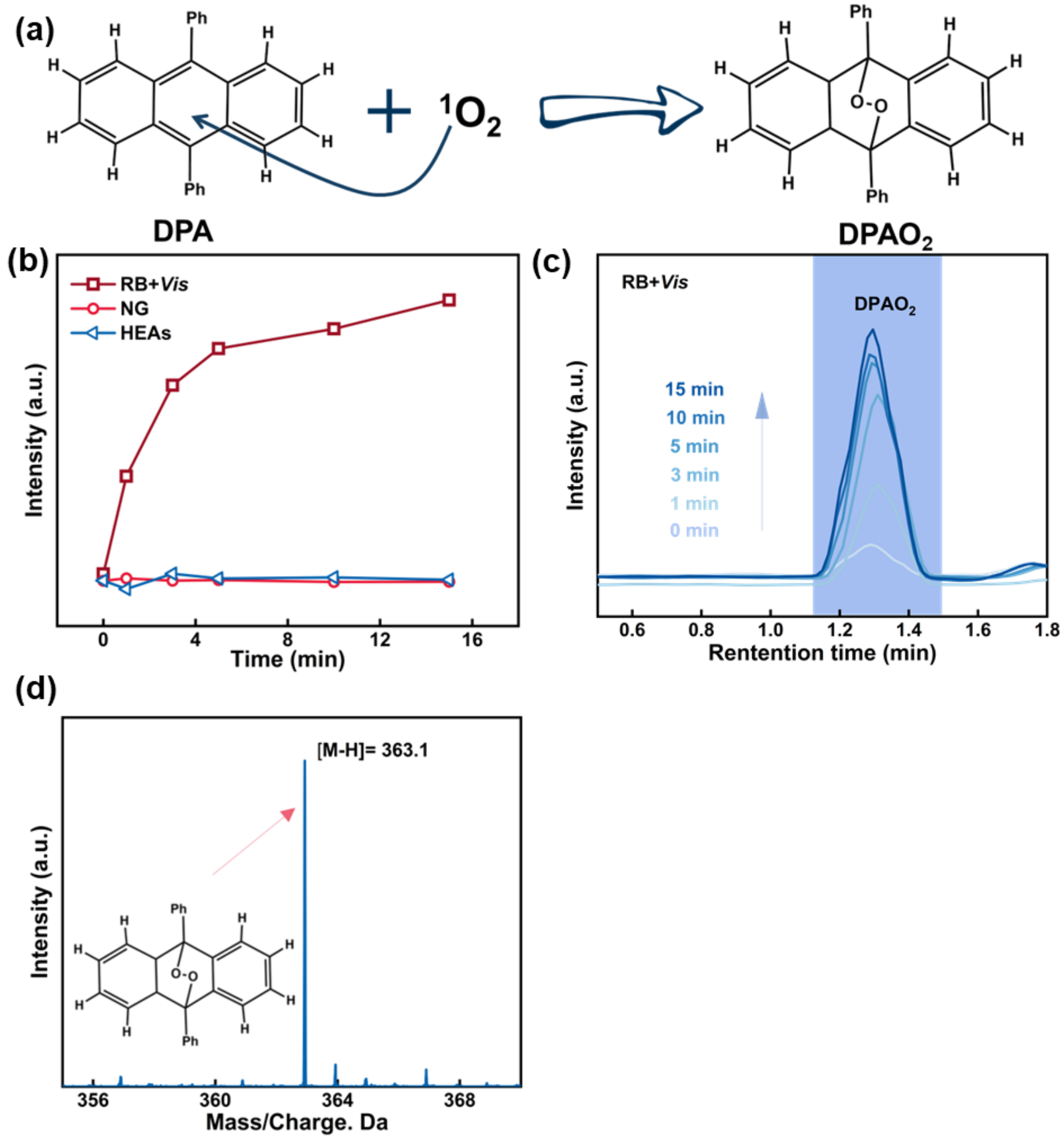

Supplementary Fig.21 (a) Schematic diagram of the principle of DPA probe and singlet oxygen generation for the target product $DPAO_2$. (b) the signal intensity of $DPAO_2$ in the different systems. (c) the signal intensity of $DPAO_2$ in RB + *vis* system. (d) the mass spectra of $DPAO_2$. Dosage: $[DPA]_0$: 0.05mM, PMS: 0.25 mM, reaction solution: 50 mL, catalyst: 0.1 g L^{-1}, reaction time: 15 min.

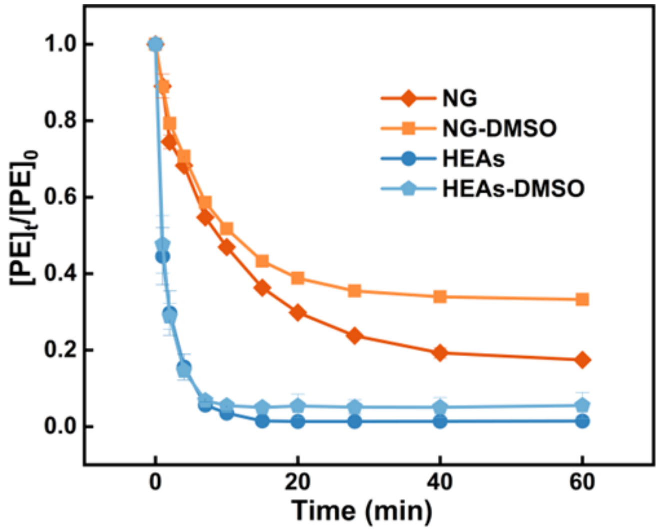

Supplementary Fig.22 Quenching effects by DMSO. Error bars represent the standard deviation, obtained by repeating the experiment three times. Dosage: $[Phenol]_0$: 0.1 mM, PMS: 0.25 mM, $[DMSO]_0$: 2.5 mM, reaction solution: 50 mL, catalyst: 0.1 g L^{-1}.

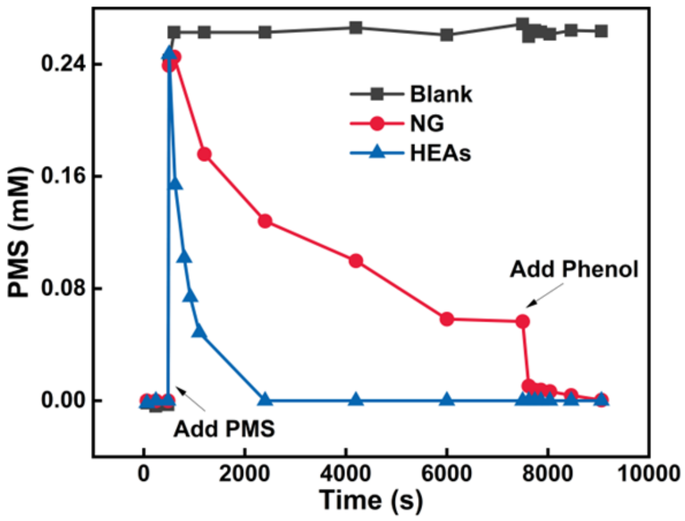

Supplementary Fig.23 The variation in PMS concentration during the OCPT experiment for HEAs-GCE and NG-GCE electrodes.

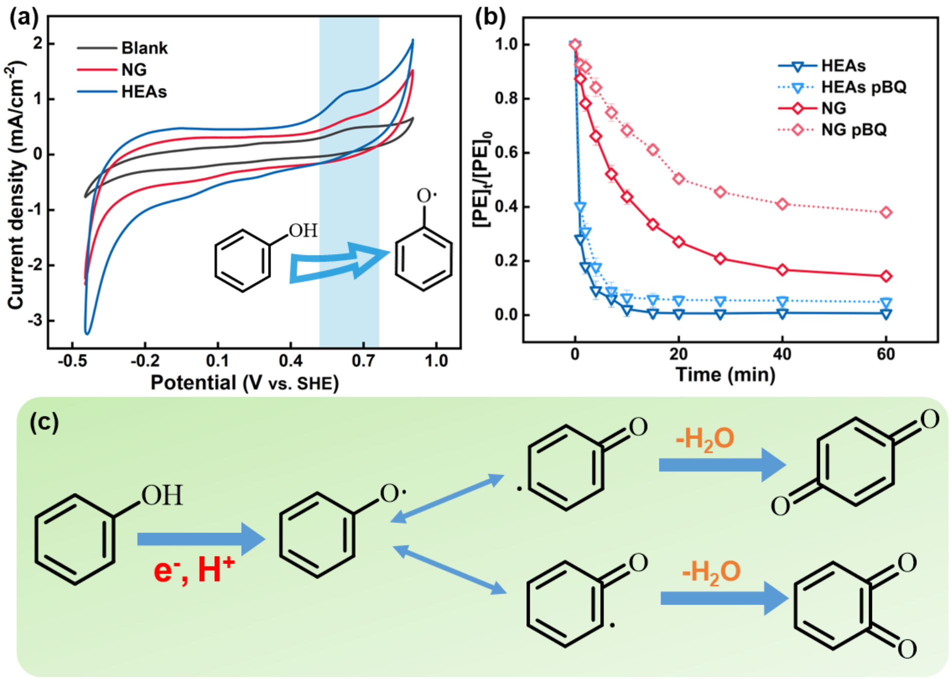

Supplementary Fig.24 (a) CV curve measurements on HEAs−GCE and NG−GCE electrode (the blue shade represents the signal of oxidation potential of phenol). (b) Quenching effects by pBQ. Error bars represent the standard deviation, obtained by repeating the experiment three times. (c) Electrochemical reaction process of phenol in CV. Dosage: $[Phenol]_0$: 0.1 mM, PMS: 0.25 mM, $[pBQ]_0$: 2.5 mM, reaction solution: 50 mL, catalyst: 0.1 g L^{-1}.

In the CV experiment, the current density generated by HEAs for the oxidation of phenol is significantly higher than that of NG, once again demonstrating that HEAs exhibit a stronger electron transfer capability than NG during the oxidation of phenol. According to the literature, the oxidation peak at around 0.64 V corresponds to the oxidation of phenol to generate phenoxy radicals, which further react to generate p-benzoquinone. This clarifies the influence of using p-benzoquinone as a quencher on the equilibrium kinetics of the electron transfer process, subsequently affecting the degradation efficiency of phenol.

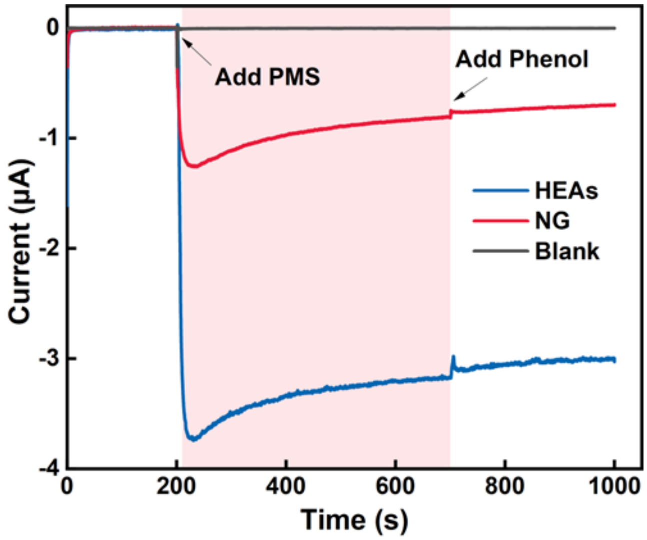

Supplementary Fig.25 Chronoamperometry curve measurements on HEAs−GCE and NG−GCE electrode.

With the addition of PMS, the current intensity increases, further indicating that in the presence of HEA and NG (electron transfer mediators), electron transfer between phenol (electron donor) and PMS (electron acceptor) can occur. The current in the HEAs-PMS* system is significantly higher than in the NG-PMS* system, highlighting the superior electron transfer capability of HEAs over NG.

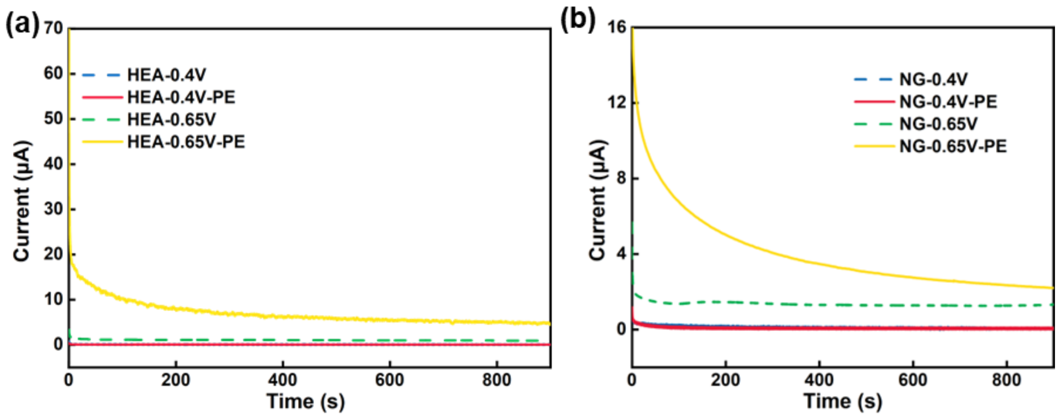

Supplementary Fig.26 The current−time curves with different potentials on the (a) HEAs−GCE electrode and (b) NG−GCE electrode.

At an equilibrium potential of +0.40 V, there is no significant current difference between HEAs-GCE and NG-GCE electrode systems with or without phenol, as the applied potential is below phenol's oxidation potential. However, when the applied potential exceeds the oxidation potential and reaches the equilibrium potential of HEAs-PMS* (+0.65 V), the current in the HEAs-GCE electrode is significantly higher than that in the NG-GCE electrode system. This indicates that the equilibrium potential of HEAs-PMS* is significantly higher than that of NG-PMS* and surpasses the oxidation potential of phenol, greatly facilitating the electron transfer process.

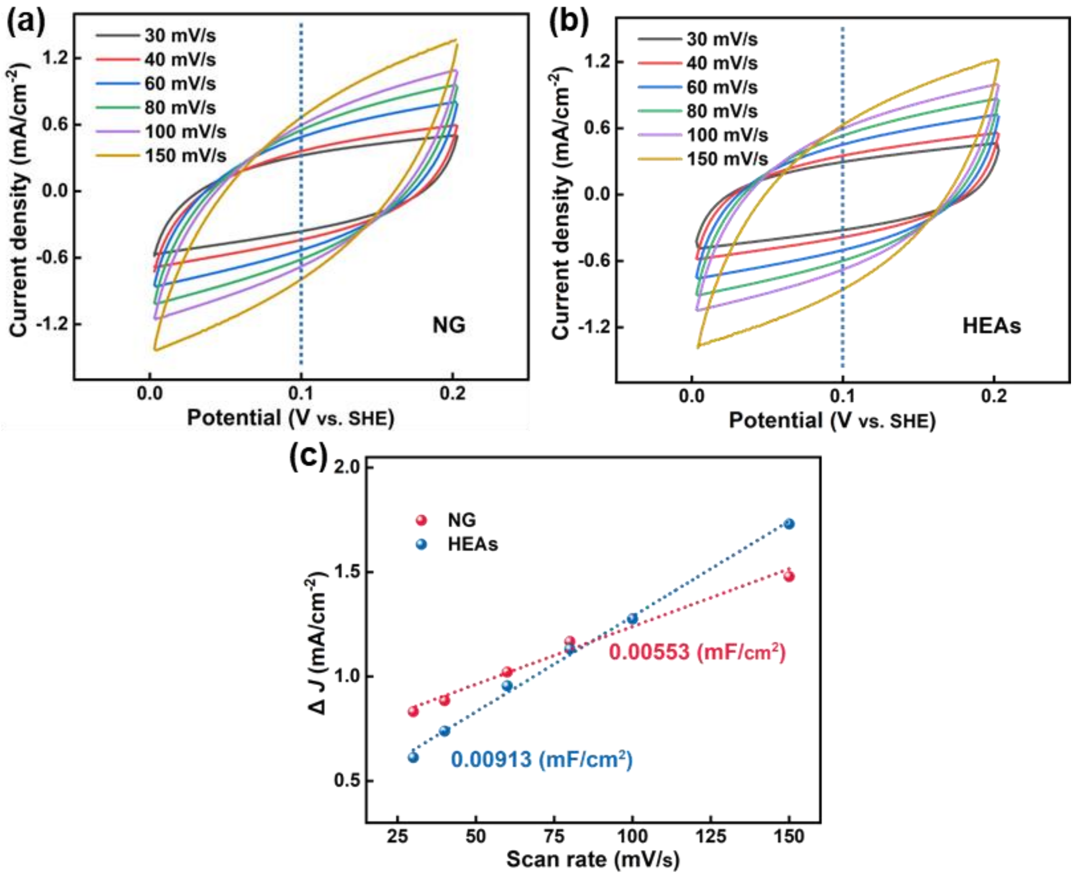

Supplementary Fig.27 CV curves for (a) NG and (b) HEAs at different scan rates from 30 to 150 mV s^{-1}, respectively. (c) Calculated C_{dl} of HEAs and NG.

To better comprehend the enhanced activity of PMS, the ECSA was estimated from the electric double-layer capacitances. HEAs exhibited a normalized capacitance 1.65 times higher than that of NG. The higher double-layer capacitance of HEAs suggests a greater number of catalytic sites, contributing to the promotion of PMS oxidation reaction rates.

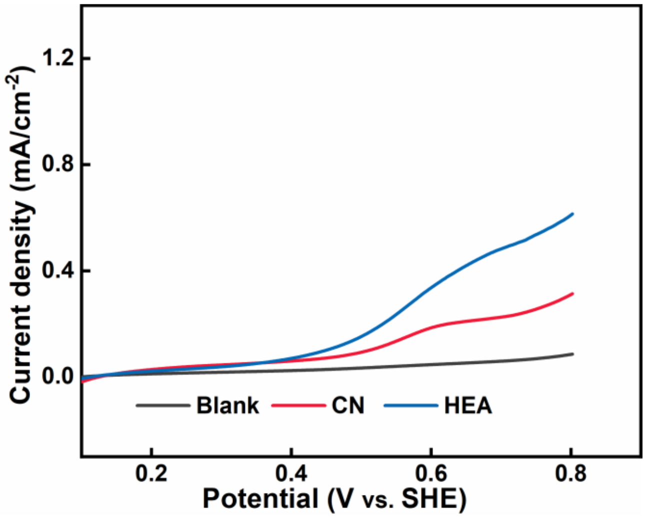

Supplementary Fig.28 LSV curves on the HEAs−GCE and NG−GCE electrodes in phenol solutions.

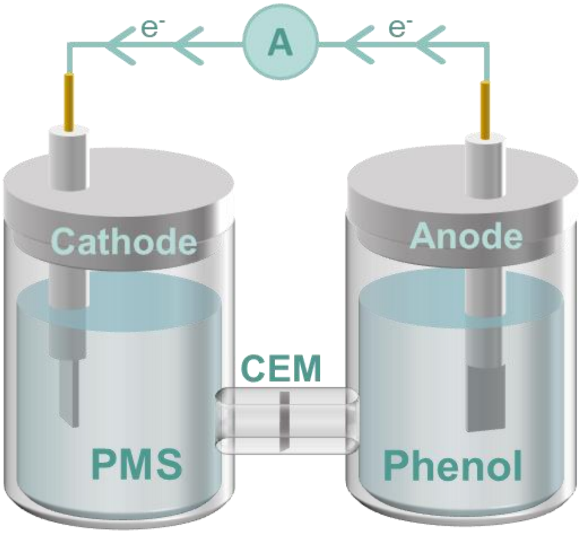

Supplementary Fig.29 The schematic diagram of galvanic reactor.

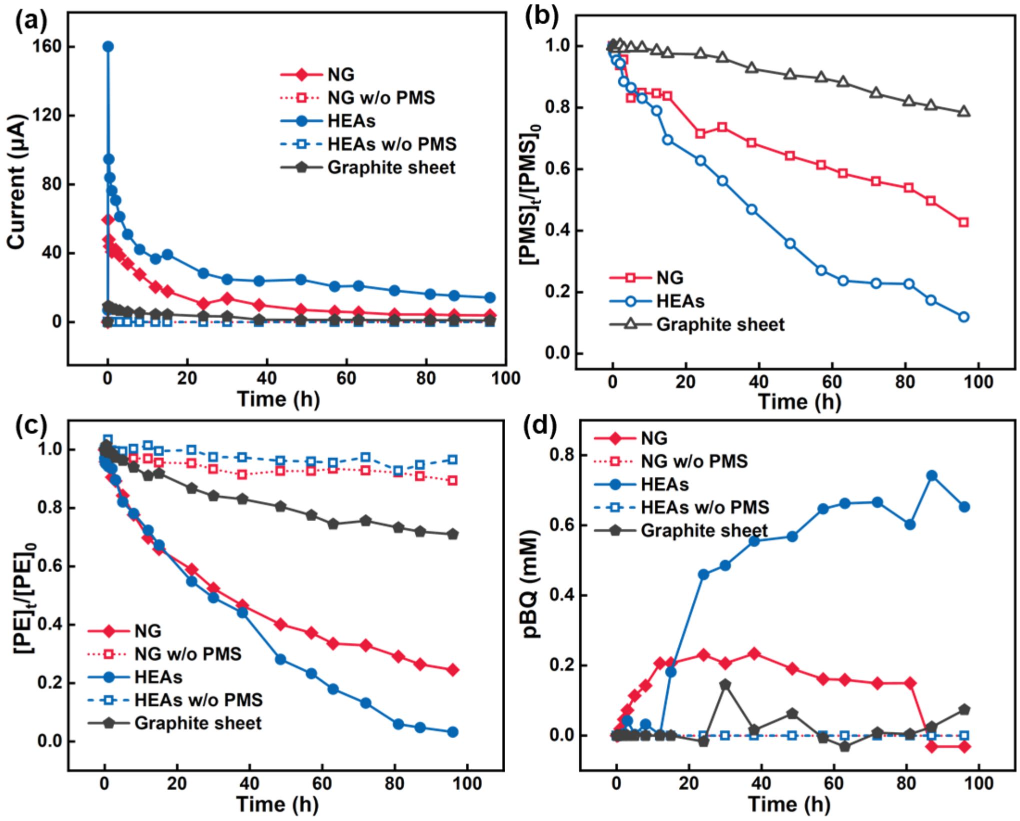

Supplementary Fig.30 (a) Current flowing from the PMS cell to the pollutants cell in galvanic reactor. (b) Decomposition rate of PMS in HEAs, NG and graphite sheet galvanic systems. (c) Degradation efficiency of phenol and (d) the concentration of pBQ in galvanic reactor anodic chamber.

The removal rate of phenol and the variations in the generation of pBQ both indicate that HEAs exhibit faster kinetics compared to NG. This once again affirms the superior activation capability of HEAs towards PMS.

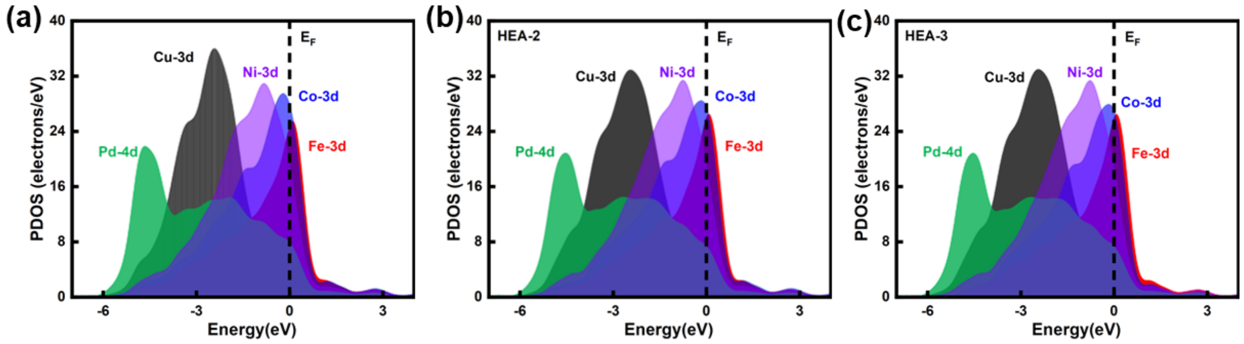

Supplementary Fig.31 (a) The PDOS of applied HEAs model in our work. (b)-(c) The PDOS of the different HEAs structures with slightly varied stoichiometry.

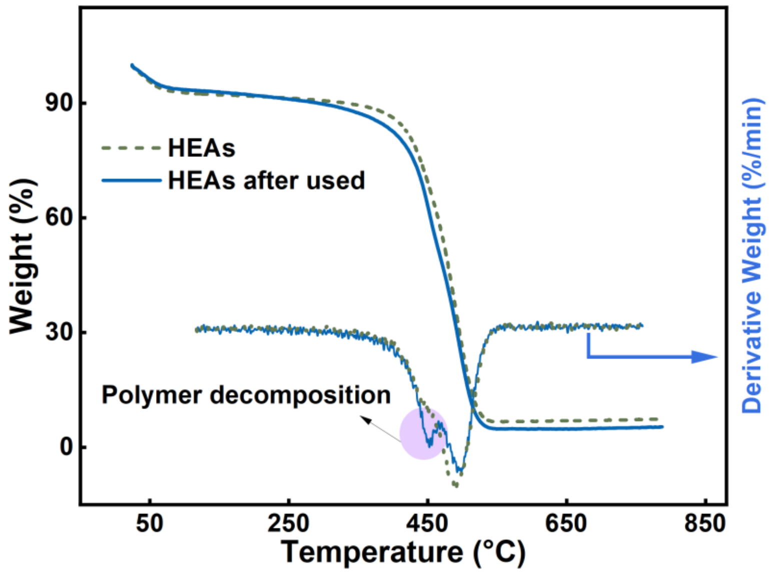

Supplementary Fig.32 (a) The TGA/DTG curves in air of HEAs and HEAs after used.

The derivative of thermogravimetric curves shows that HEAs alone has only one peak, and after activating PMS to degrade phenol, there are obviously two kinds of substances that are thermally decomposed, one is the carbon-based catalyst is thermally decomposed in air, and the other is the phenolic polymerization products on the surface of HEAs are decomposed. The mechanism of phenol polymerization on HEAs surface was verified again.

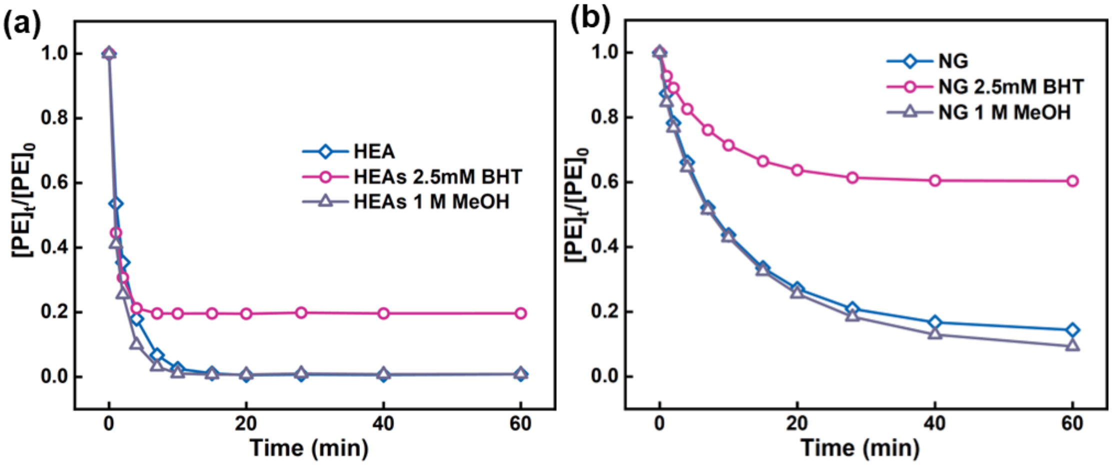

Supplementary Fig.33 Quenching effects by MeOH and BHT scavengers of (a) HEAs and (b) NG. Dosage: $[Phenol]_0$: 0.1 mM, PMS: 0.25 mM, reaction solution: 50 mL, catalyst: 0.1 g L^{-1}.

BHT was used to quench the polymerization activity of phenoxy radicals, and it was found that the oxidation rate of phenol was significantly reduced, which once again confirmed that polymerization plays an important role in the oxidation process of phenol. due to the solubility of BHT, it is not possible to completely terminate the reactions in the HEAs-PMS and NG-PMS systems.

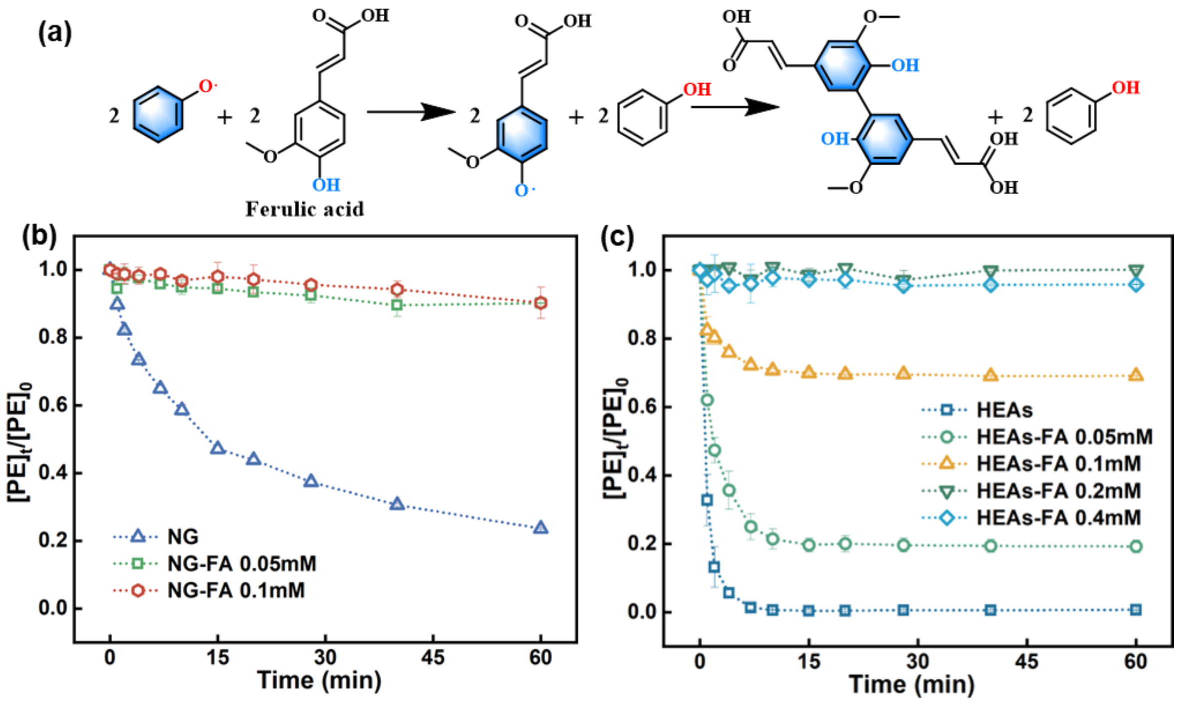

Supplementary Fig.34 (a) The schematic illustration of phenoxyl radicals quenching by FA. The effect of FA on phenol degradation of NG-PMS (b) and HEAs-PMS (c) system. Error bars represent the standard deviation, obtained by repeating the experiment two times. Dosage: $[Phenol]_0$: 0.1 mM, PMS: 0.25 mM, reaction solution: 50 mL, catalyst: 0.1 g L^{-1}.

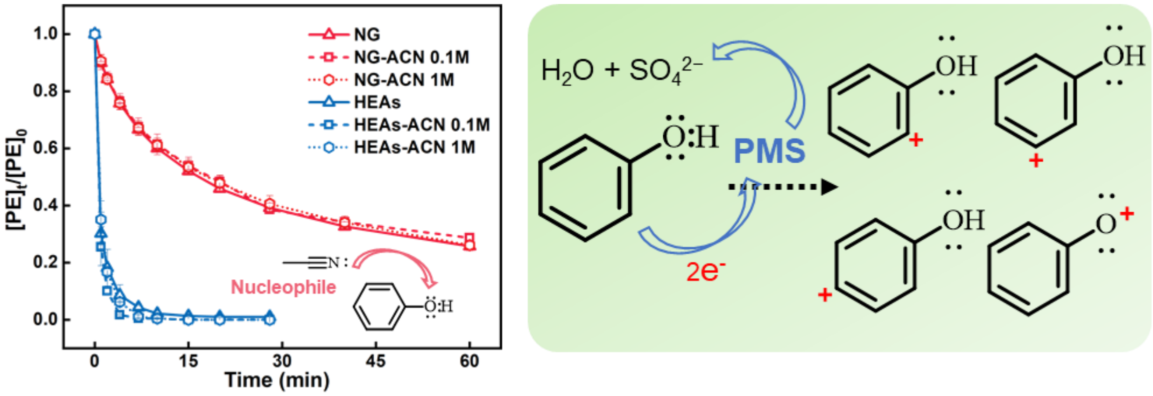

Supplementary Fig.35 The competitive inhibitory effect of acetonitrile on PhOH removal in NG-PMS and HEAs-PMS systems, and a schematic diagram illustrating the surface double electron transfer process of DOTP. Error bars represent the standard deviation, obtained by repeating the experiment three times.

Since the electrophilic phenoxonium ion can be scavenged by a stronger nucleophile (acetonitrile), the reaction of PhOH should be inhibited by acetonitrile. Beaker experiments adding acetonitrile explored this effect, and no decrease in removal rates was observed, proving that the phenoxonium ion intermediate does not exist in the HEAs-PMS and NG-PMS systems.

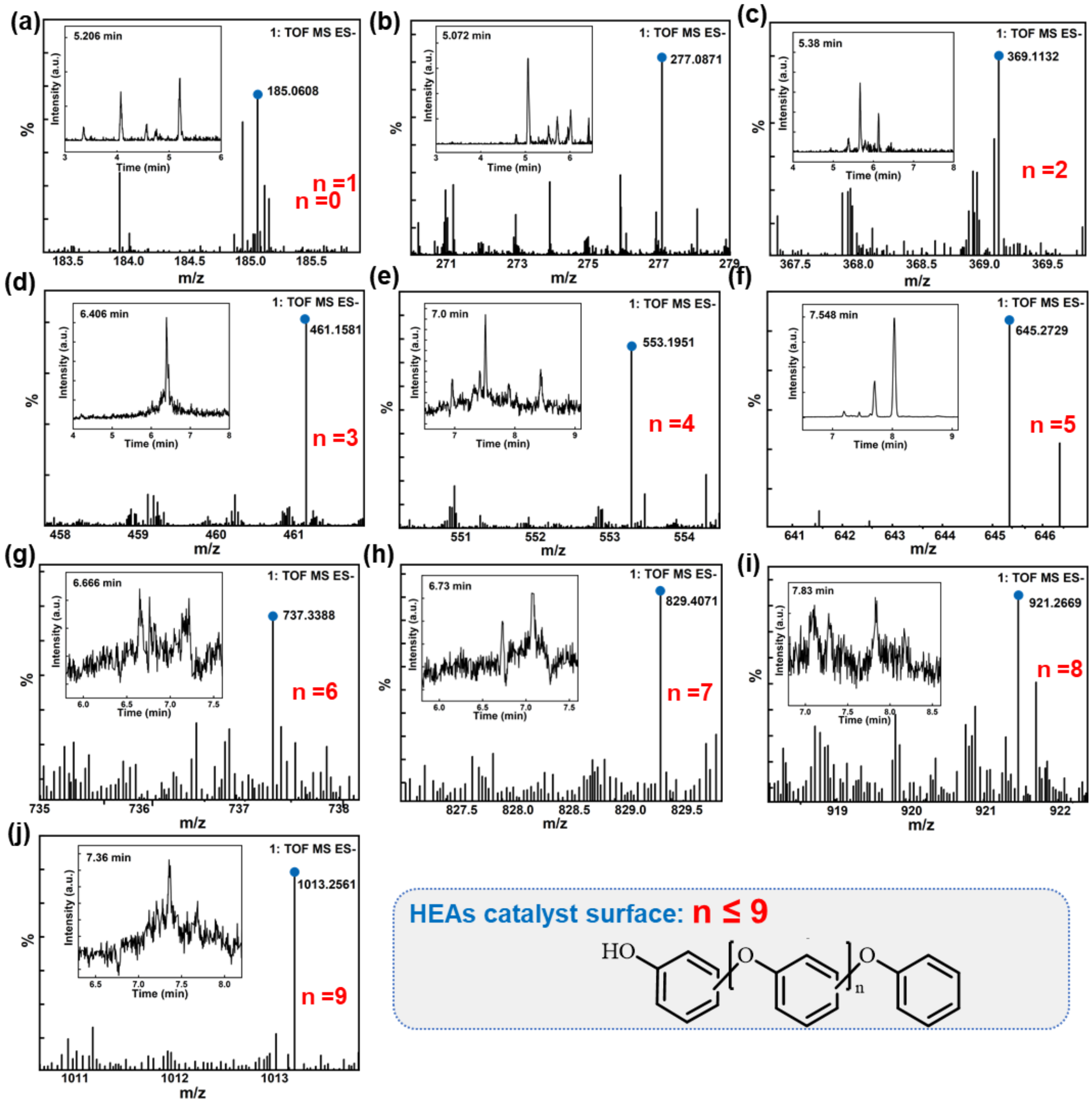

(a)
5.206 min
1: TOF MS ES-
185.0608
n =1
n =0
(b)
5.072 min
1: TOF MS ES-
277.0871
(c)
5.38 min
1: TOF MS ES-
369.1132
n =2
(d)
6.406 min
1: TOF MS ES-
461.1581
n =3
(e)
7.0 min
1: TOF MS ES-
553.1951
n =4
(f)
7.548 min
1: TOF MS ES-
645.2729
n =5
(g)
6.666 min
1: TOF MS ES-
737.3388
n =6
(h)
6.73 min
1: TOF MS ES-
829.4071
n =7
(i)
7.83 min
1: TOF MS ES-
921.2669
n =8
(j)
7.36 min
1: TOF MS ES-
1013.2561
n =9
Intensity (a.u.)
Time (min)
%
m/z
HEAs catalyst surface: n ≤ 9
HO
O
O
n

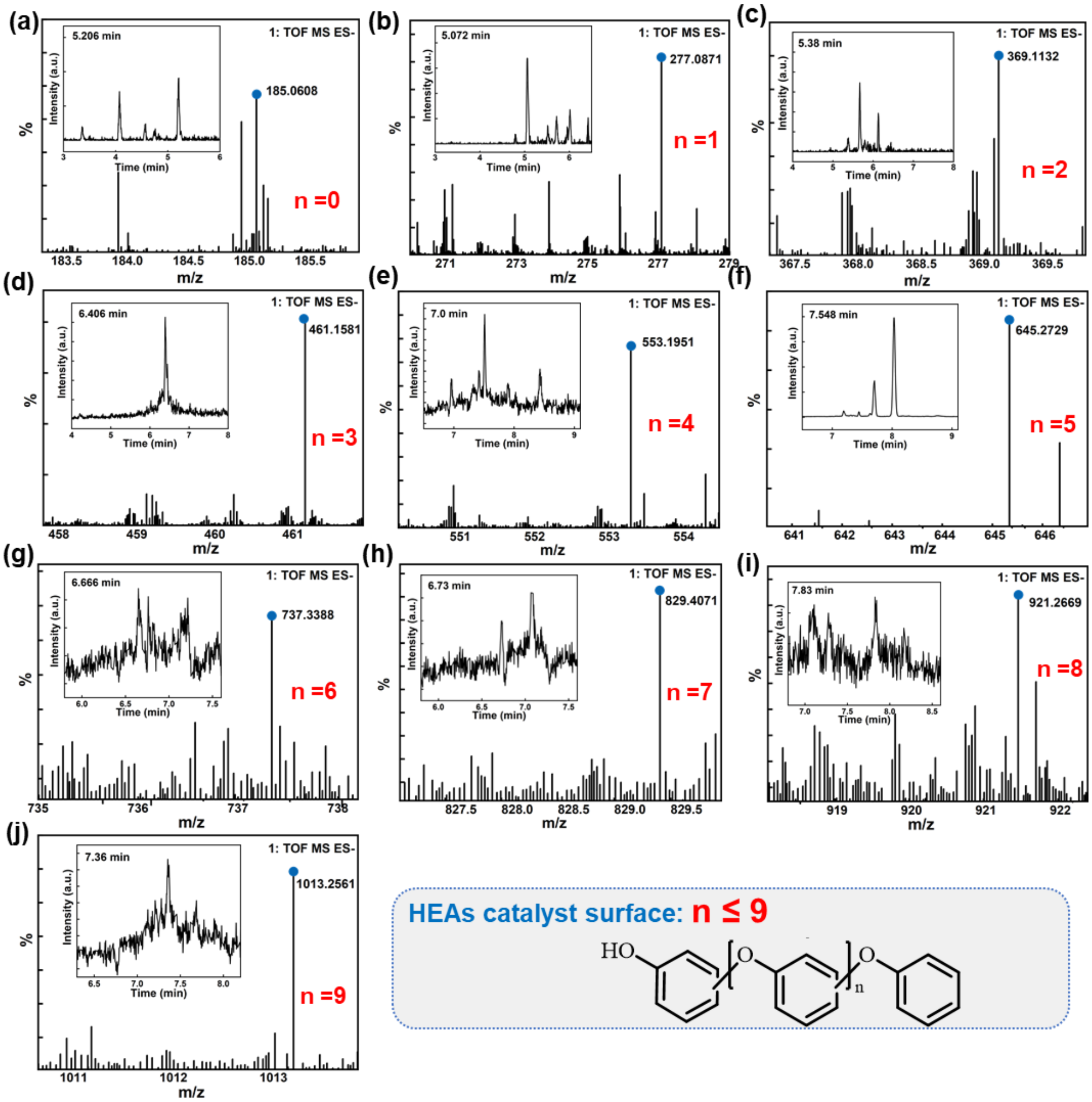

Supplementary Fig.36 UPLC–QTOF-MS molecular ion mass spectra of the chromatographic peaks of phenol oxidation polymerization products on the HEAs catalyst surface, along with their corresponding chromatograms (inset figure).

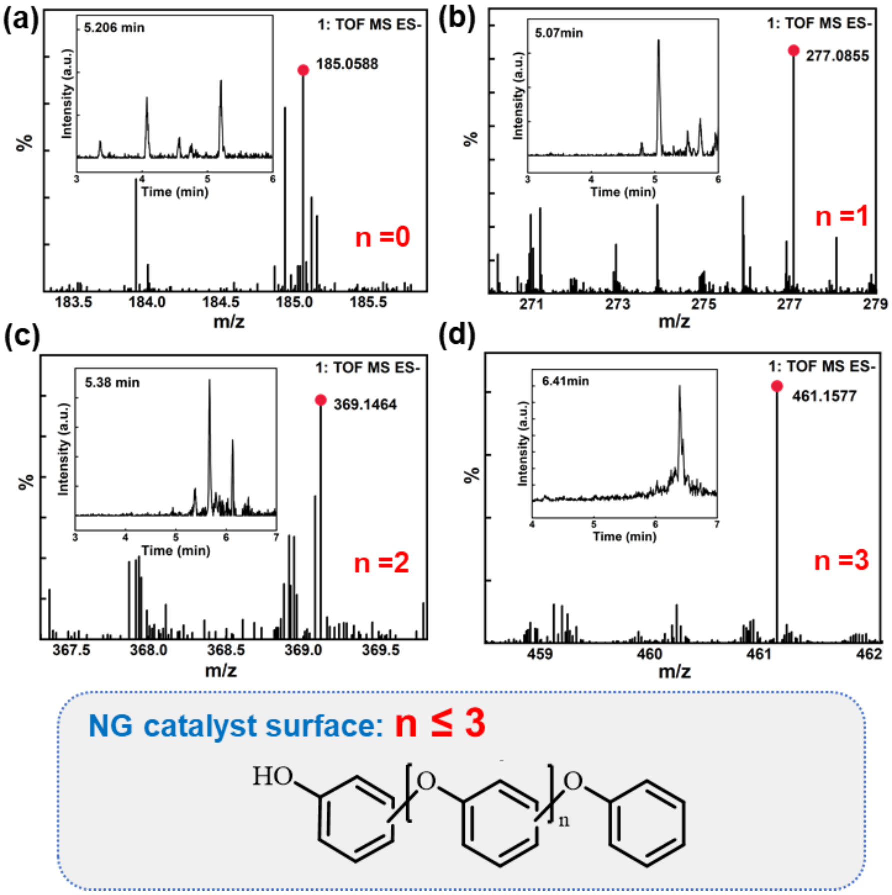

Supplementary Fig.37 UPLC–QTOF-MS molecular ion mass spectra of the chromatographic peaks of phenol oxidation polymerization products on the NG catalyst surface, along with their corresponding chromatograms (inset figure).

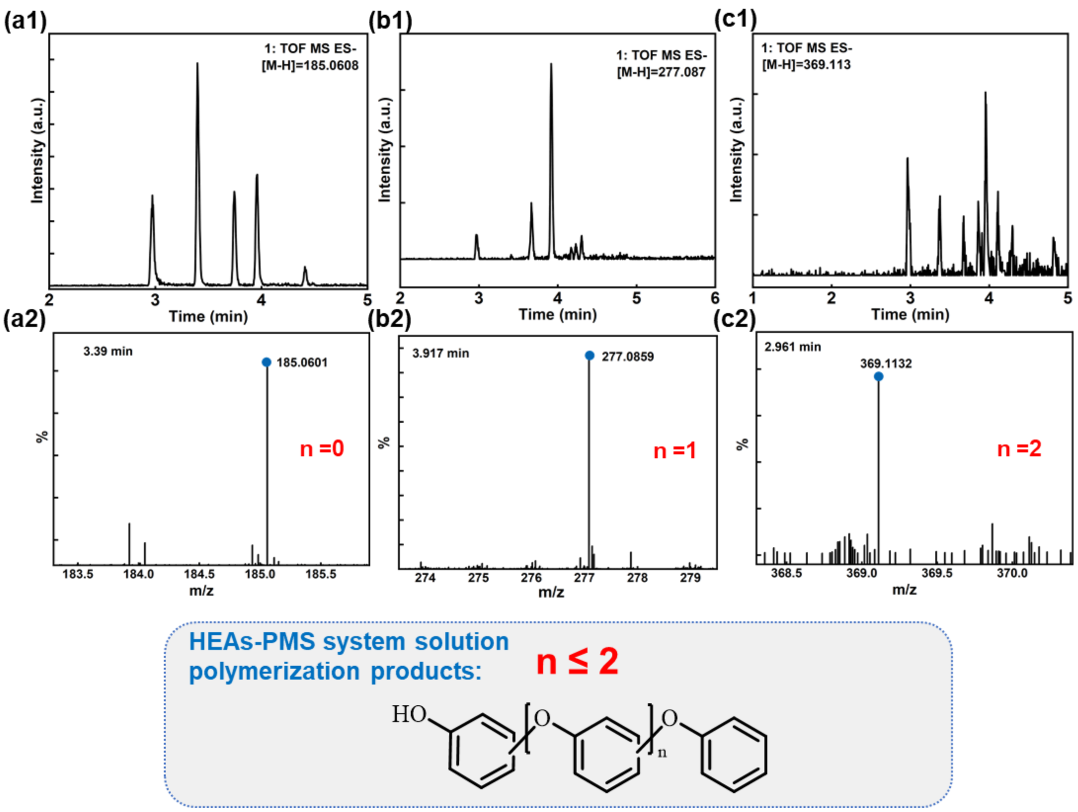

Supplementary Fig.38 UPLC–QTOF-MS chromatograms of phenol oxidation polymerization products in the HEAs–PMS system solution, and their corresponding molecular ion mass spectra of the chromatographic peaks.

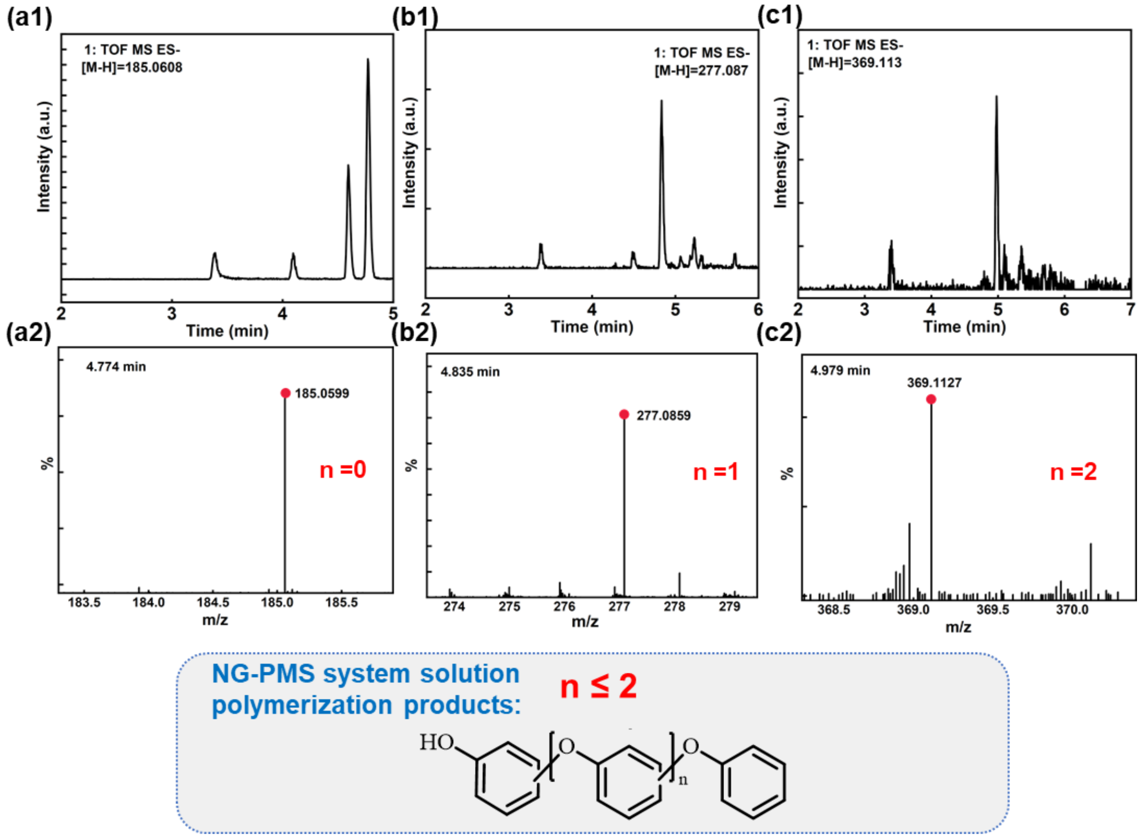

Supplementary Fig.39 UPLC–QTOF-MS chromatograms of phenol oxidation polymerization products in the NG–PMS system solution, and their corresponding molecular ion mass spectra of the chromatographic peaks.

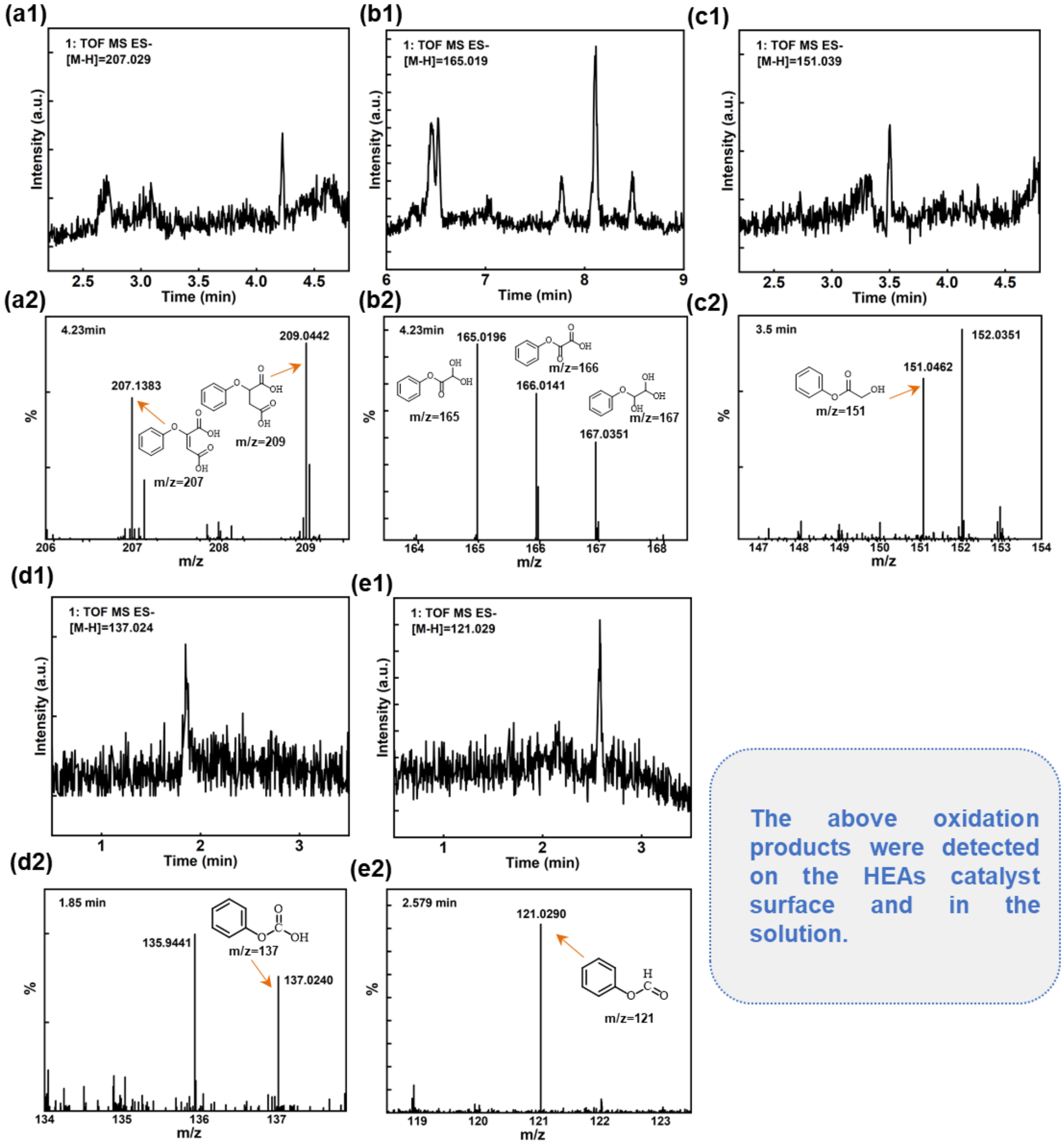

Supplementary Fig.40 UPLC–QTOF-MS chromatograms of phenol oxidation products in the HEAs–PMS system (detected on both catalyst surface and solution), and their corresponding molecular ion mass spectra of the chromatographic peaks.

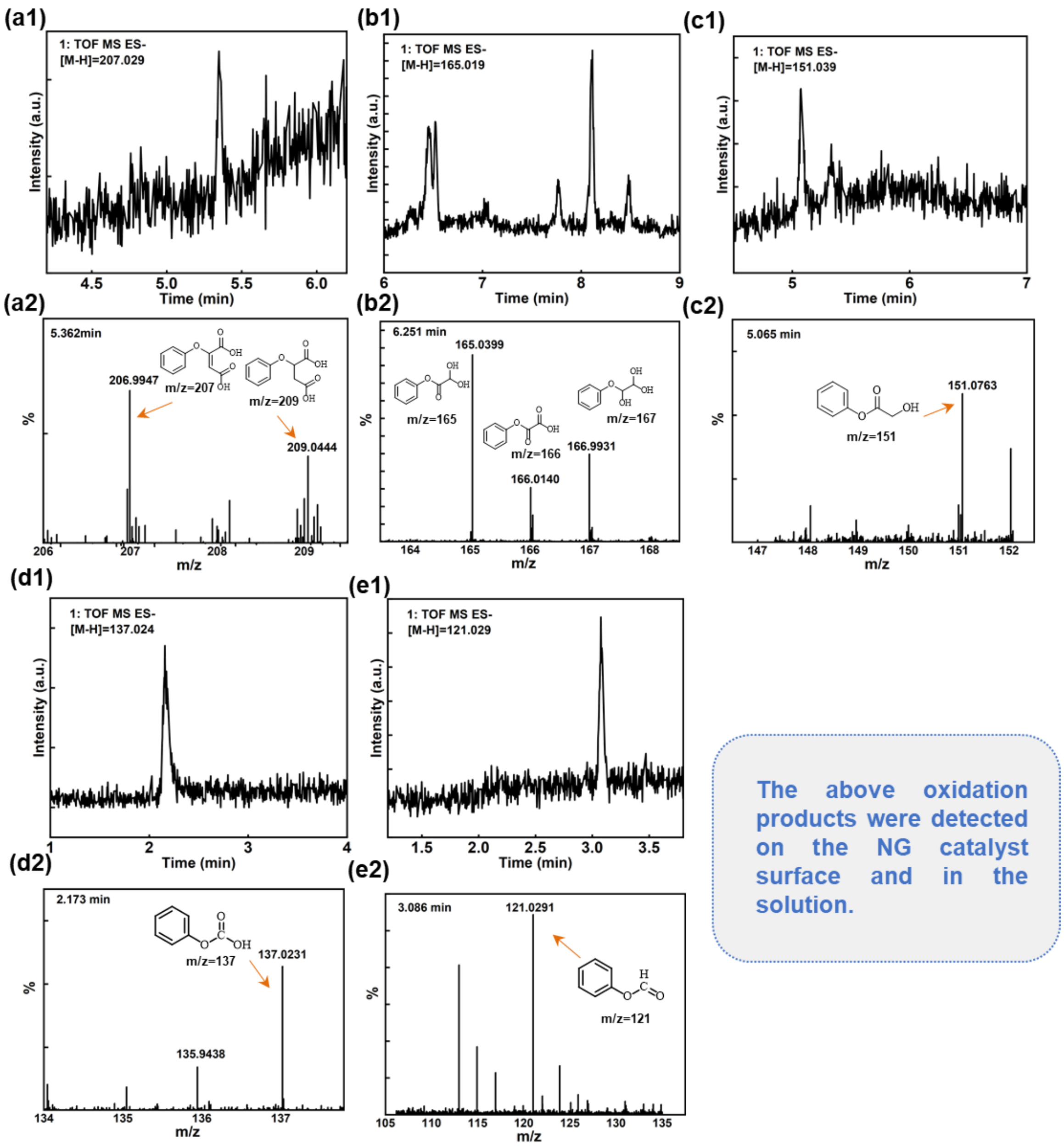

Supplementary Fig.41 UPLC–QTOF-MS chromatograms of phenol oxidation products in the HEAs–PMS system (detected on both catalyst surface and solution), and their corresponding molecular ion mass spectra of the chromatographic peaks.

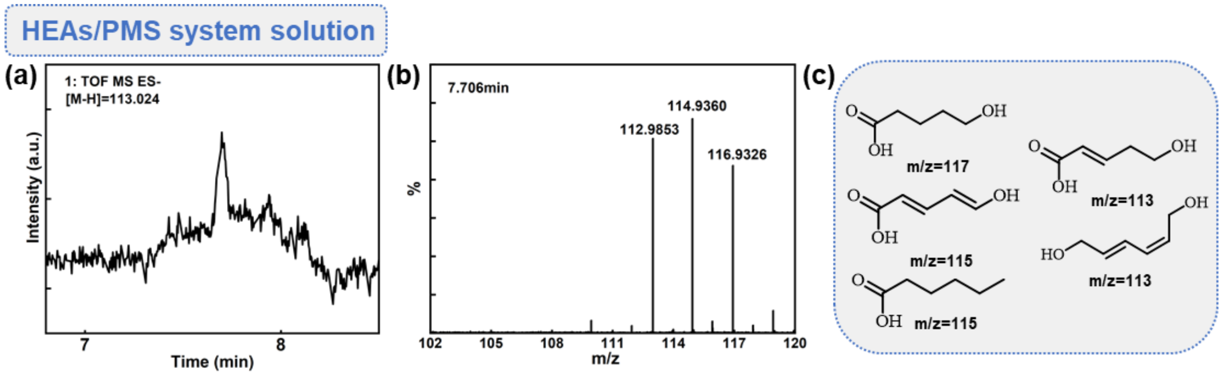

Supplementary Fig.42 UPLC–QTOF-MS chromatograms of phenol oxidation products in the HEAs–PMS system solution, and their corresponding molecular ion mass spectra of the chromatographic peaks.

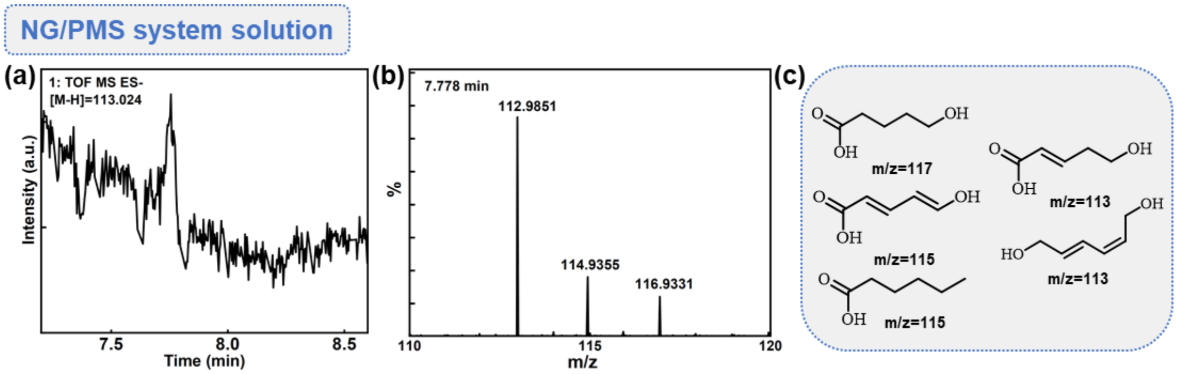

Supplementary Fig.43 UPLC–QTOF-MS chromatograms of phenol oxidation products in the NG–PMS system solution, and their corresponding molecular ion mass spectra of the chromatographic peaks.

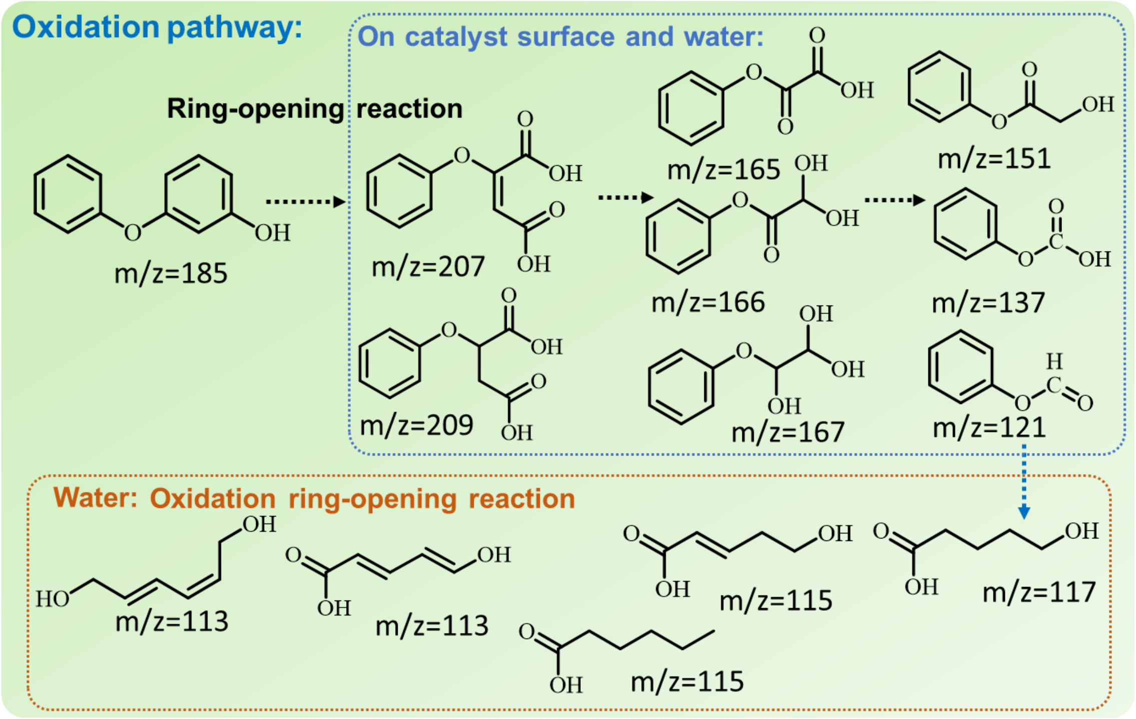

Supplementary Fig.44 Proposed reaction pathways of oxidative ring-opening pathways in aqueous solution.

Taking dimers as an example, further analysis of the mineralization degradation pathway was proposed according to the product analysis. As shown in *SI Appendix*, Fig. S38-S42, oxidation products resulting from the ring-opening reaction of dimers (*m/z* =121-209) were detected on the catalyst surface and in the solution, while some small-molecule and fully oxidized products (*m/z* <117) were only detected in the solution (Fig. 4*D*). Drawing insights from the aforementioned analysis, a proposed conversion pathway for phenol is outlined, emphasizing the polymerization process as a prevalent yet previously overlooked non-radical pathway for PMS oxidation.

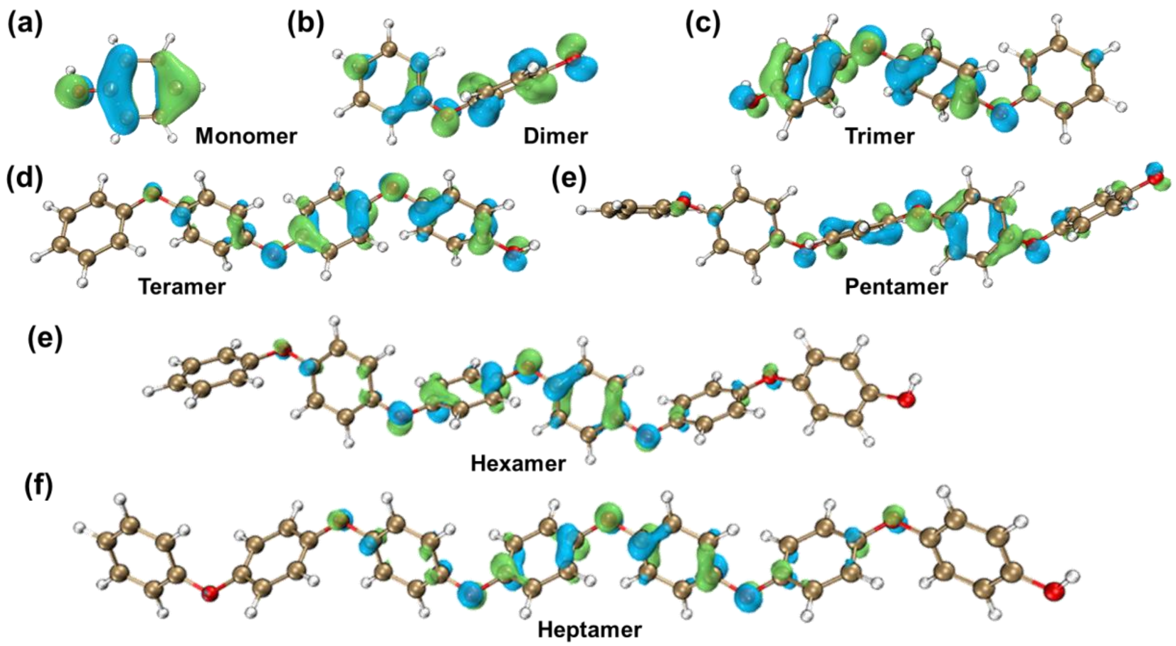

Supplementary Fig.45 a-f Electron Density Difference diagrams for different units polymerization-degree phenol. The brown, white, and red balls represented C, H, and O atoms, respectively. The charge accumulation is shown as the green region, and the charge depletion is shown as the cyan region

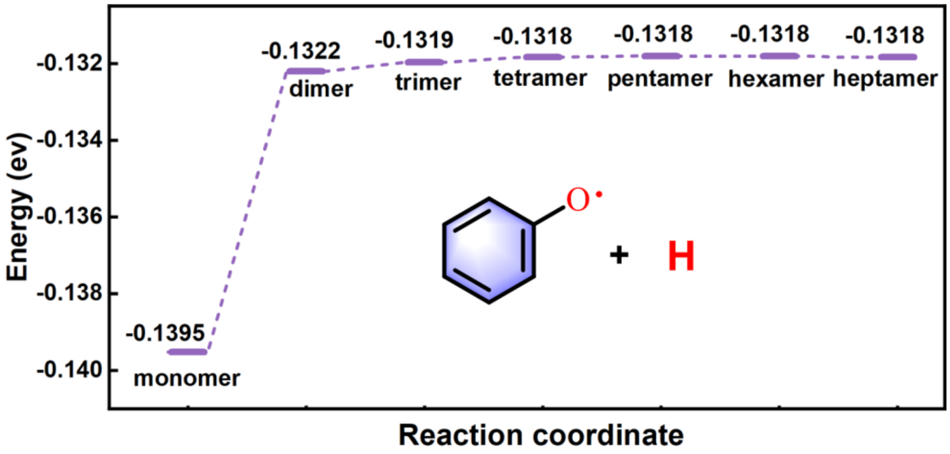

Supplementary Fig.46 Thermodynamic potential energy curves for different units polymerization-degree phenol radical generation.

The dissociation energy in the formation process of phenoxy radicals was determined through adsorption energy calculations using DFT quantum chemical methods, further validating the previously proposed mechanism. The results show that as the degree of polymerization increases, the thermodynamic free energy of the reaction gradually decreases. This implies that reactants with a higher degree of polymerization require a lower potential difference to oxidize into phenoxy radicals, facilitating the formation of the corresponding polymer products. Simultaneously, as the degree of polymerization increases, the dissociation energy gradually stabilizes, indicating that the energy barrier for polymer formation is no longer associated with the generation of organic radicals, but rather with other factors within the reaction system.

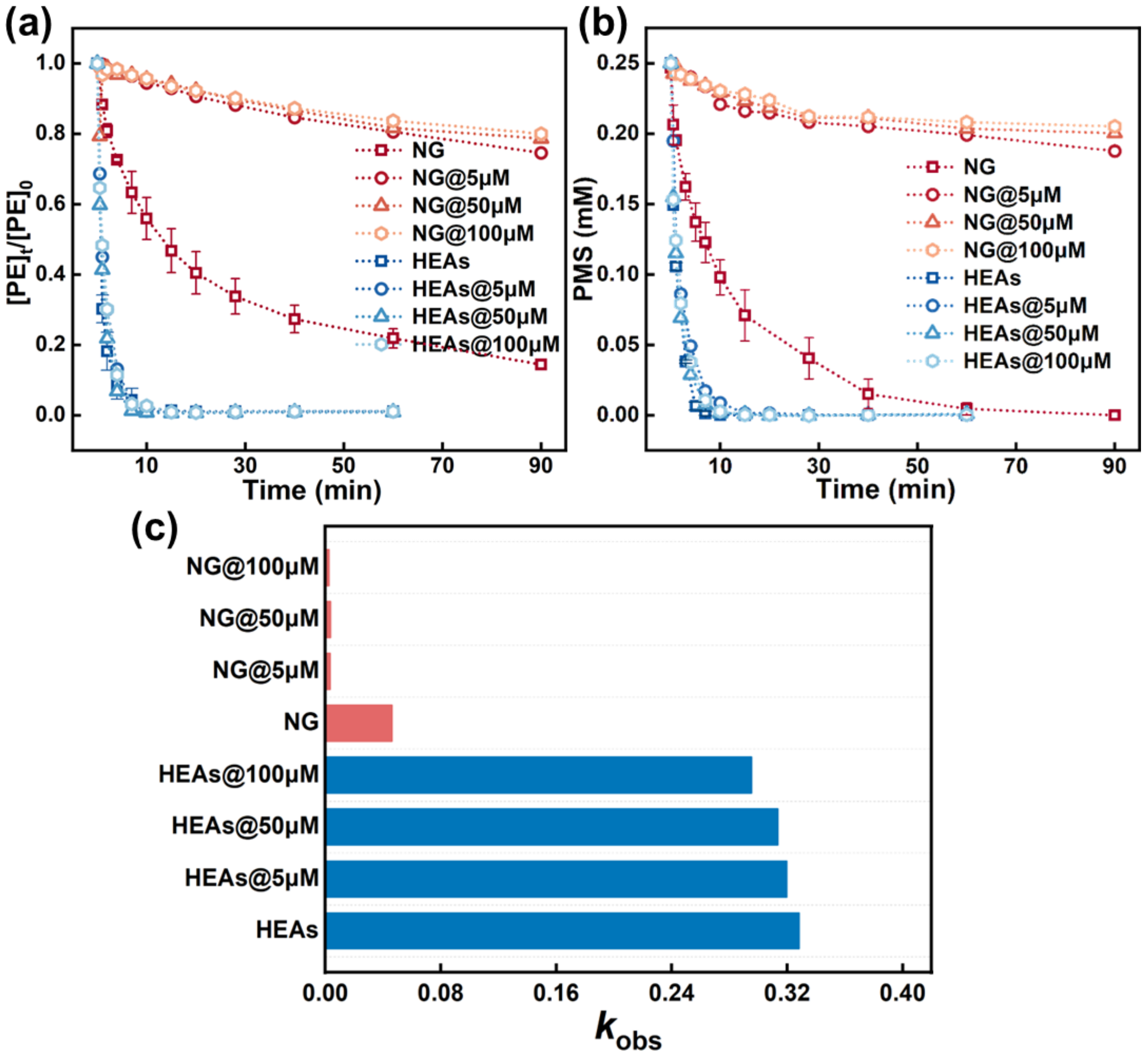

Supplementary Fig.47 (a) Degradation efficiency of phenol with different prepolymerized products on NG and HEAs surfaces as catalysts. (b) Decomposition rate of PMS in degradation systems with different prepolymerized products on HEAs and NG surfaces. (c) Degradation apparent rate constants of phenol with different prepolymerized products on NG and HEAs surfaces as catalysts. Error bars represent the standard deviation, obtained by repeating the experiment three times. Dosage: $[Phenol]_0$: 0.1 mM, PMS: 0.25 mM, reaction solution: 50 mL, catalyst: 0.1 g L^{-1}.

Phenol polymers with different equivalents of 5 μm, 50 μm, and 100 μm were pre-polymerized on the surfaces of HEAs and NG through a 0.25 mM PMS-triggered polymerization reaction, denoted as NG@5 μm, HEAs@5 μm, NG@50 μm, HEAs@50 μm, NG@100 μm and HEAs@100 μm, respectively.

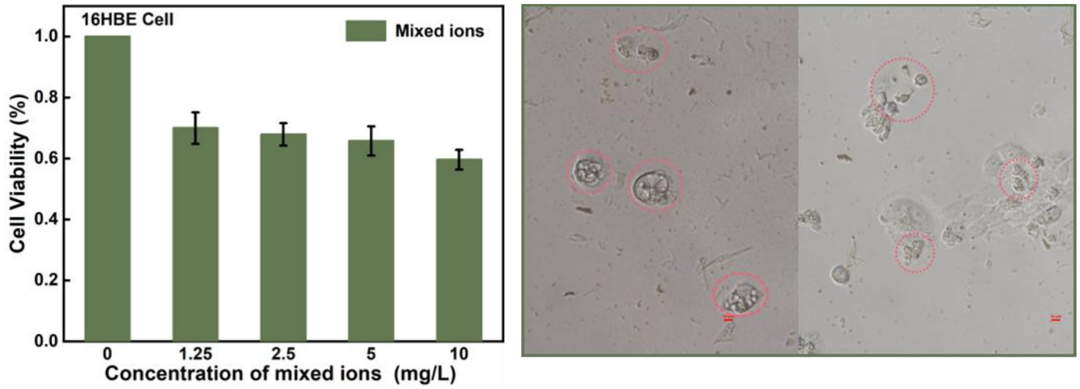

Supplementary Fig.48 Cell viability of 16HBE under different concentrations of mixed ions and cells in the culture medium were observed under a microscope. Cell culture time: 48 h. Error bars represent the standard deviation, obtained by repeating the experiment three times. The red circle indicates cell morphology disruption, signifying cell death.

Exposed to five types of mixed metal ions, the cell viability of 16HBE is significantly inhibited by the mixed ions. In the microscopic field, the cell morphology is noticeably disrupted, and a substantial number of cells undergo cell death.

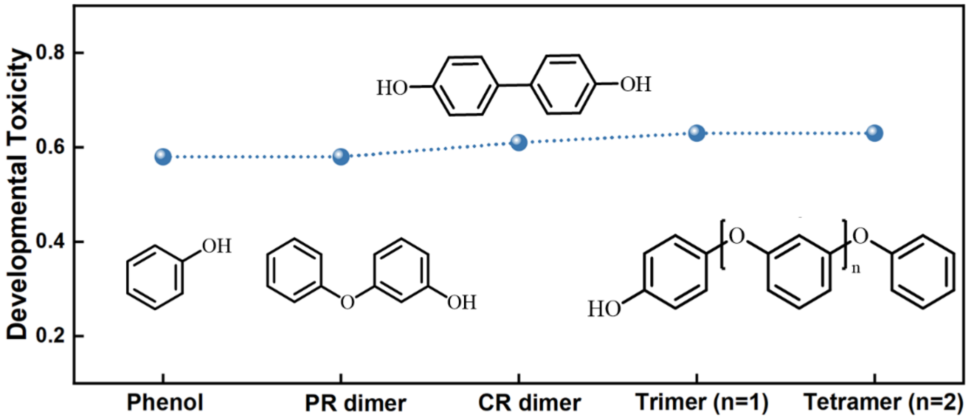

Supplementary Fig.49 Developmental toxicity at varying degrees of polymerization calculated using the Toxicity Estimation Software Tool (TEST).

The results showed that developmental toxicity remained relatively stable, with a slight increasing trend as the degree of polymerization increased. These findings underscore the importance of recovering polymeric products when removing organic compounds through polymerization-driven pathways in heterogeneous oxidation processes. Consequently, developing efficient recovery methods and relevant technologies is essential.

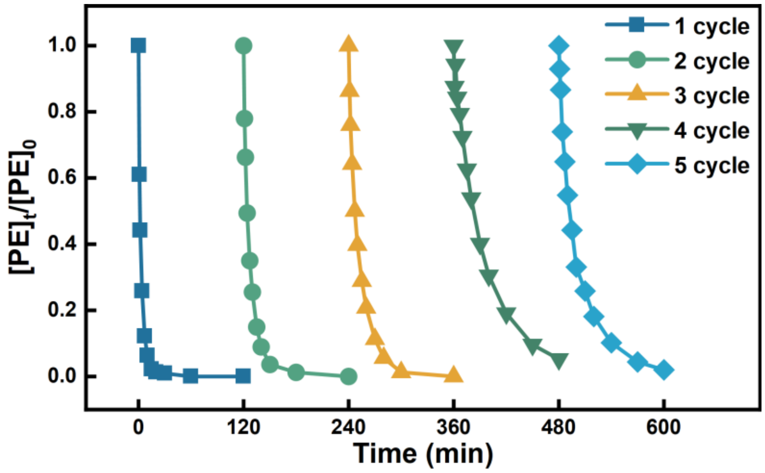

Supplementary Fig.50 Cycle experiment for HEAs. Dosage: [Phenol]0: 0.1 mM, PMS: 0.25 mM, reaction solution: 50 mL, catalyst: 0.1 g L^{-1}.

Cycle experiments with HEAs revealed that the catalyst exhibits excellent stability during the cyclic degradation of phenol, achieving a removal rate of 95% within the first five cycles. This observation further indicates that HEAs activate PMS through HEA NPs, with polymerization products mainly forming on the graphite surface. This enables continued rapid activation of PMS and allows for a sustained thermodynamically favorable polymerization process, resulting in further phenol polymerization and the production of high molecular weight products. These results effectively explain why HEAs produce higher polymerization products compared to NG.

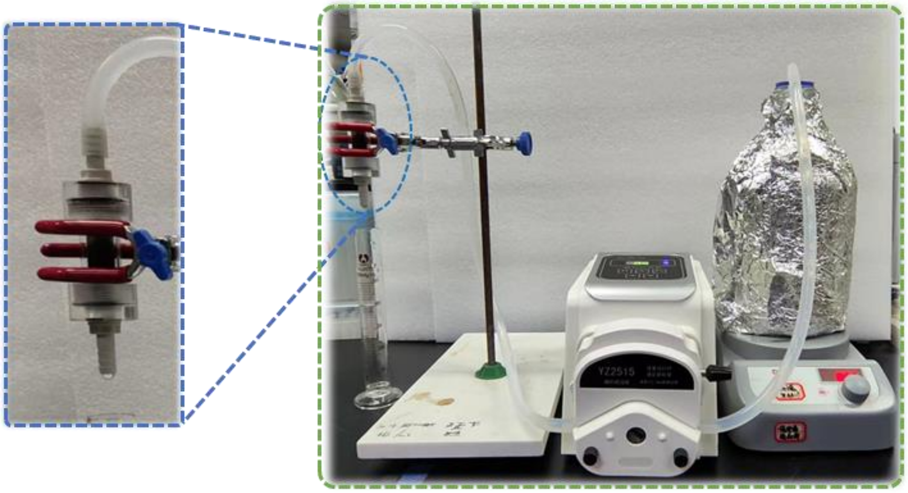

Supplementary Fig.51 Physical diagram of the continuous-flow reactor.

Supplementary Table 1. Atomic ratios of HEAs characterized by ICP.

sample	Cu	Pd	Fe	Co	Ni
Content ratios	12	11	10	11	12

Supplementary Table 2. The CHON element ratios of HEAs and NG characterized by Organic Element Analyze.

Sample	C	H	O	N
HEAs	71.472	2.581	8.419	8.075
NG	72.132	2.747	7.924	15.027

Supplementary Table 3. The concentration of metal ion in the solution of ions-PMS system. Dosage: $[Phenol]_0$: 0.1 mM, PMS: 0.25 mM L^{-1}, reaction solution: 50 mL, reaction time: 60 min.

Ion (mg L^{-1})	Cu	Pd	Fe	Co	Ni
equivalents Co^{2+}				0.49	
equivalents Cu^{2+}	0.55				
equivalents Pd^{2+}		0.875			
equivalents Fe^{3+}			0.435		
equivalents Ni^{2+}					0.525

Supplementary Table 4. The literature reports the concentration of residual metal ions in the solution of metal catalyst-PMS system and HEAs-PMS system. Dosage: [Phenol]$_0$: 0.1 mM, PMS: 0.25 mM L^{-1}, reaction solution: 50 mL, catalyst: 0.1 g L^{-1}, reaction time: 60 min.

Ion (mg L^{-1})	Cu	Pd	Fe	Co	Ni	Reference
HEAs	0.025	0.014	0.009	0.042	0.007	This study
HEAs (pH=1)	-	0.029	0.008	0.001	-	
Co Mo-DSA				0.39		1
Co–N–C				0.63		1
Co-N_3O_1/C				~0.24		2
CuSA/MXene	~0.037					3
Co-N_2/C				0.25		4
Co-NC				39.22		4
FeCo−N/C-3			~0.175	~0.16		5
C@Cu-Ni	4.72				0.5	6
NiSA@CN					0.022	7
Pd/Al_2O_3		~0.38				8
Pd_8/CMK-3		~2.32				9

"-"-indicates that it has not been detected; **"~"**-indicates that no specific data is given in the literature and is identified by the value in the figure.

Supplementary Table 5. Comparison of catalytic reactivity of HEAs catalyst with previous reported catalysts during PMS activation.

Catalyst	Catalyst dosage (g L^{-1})	Pollutant (mM)	PMS (mM)	Removal efficiency	Rate constant (min^{-1})	normalized k value [a]	Reference
HEAs	0.1	Phenol (0.1)	0.25	100% (10min)	0.328	1.312	This study
CoMo-DSA	0.3	Phenol (0.31)	0.65	100% (15min)	0.33	0.524	1
Co-N_3O_1	0.1	Phenol (0.016)	1.0	100% (20min)	~0.26	4.16×10^{-2}	2
CuSA/MXene	0.5	BPA (0.04)	2	98% (10min)	0.23	9.02×10^{-3}	3
Co-N_4	0.2	BPA (0.05)	2	~20% (5min)	0.073	1.46×10^{-3}	4
NiSA@CN	0.05	BPA (0.04)	0.65	100% (25min)	0.129	0.159	7
FeCo−N/C	0.05	Phenol (0.16)	1.3	100% (30min)	0.31	0.763	5

[a] The Specific activity (normalized k value) was calculated through dividing the observed rate constant of pollutant by the catalyst dosage and PMS concentration, followed by multiplying organic contaminant concentration.

Supplementary Table 6. Comparison of the apparent electron utilization efficiency of different systems.

Systems	[Phenol] (mM)	TOC (%)	Given e^{-} (mM) [a]	[Oxidant] (mM)	Obtained e^{-} (mM) [b]	e^{-} utilization (%) [c]
HEAs–PMS	0.1	38.11	1.067	0.25	0.5	213.4
NG–PMS	0.1*78.39%	33.43	0.734	0.25	0.5	146.7

[a] The given electron equivalent of pollutant mineralization is calculated according to the mineralization rate. Take HEAs–PMS as an example, the given e^{-} = [phenol] × TOC removal × 28 = 0.1 × 38.11% × 28 = 1.067 mM .

$C_6H_5OH + 11H_2O - 28e^{-} \rightarrow 6CO_2 + 28H^{+}$

[b] This value was calculated according to the actual dosage of oxidant (PMS or PDS). One mole of PMS accepts two moles of electrons in the reaction process. For HEAs–PMS system as an example, the e^{-} equivalence of oxidant was calculated from: [PMS]×2 = 0.25×2 = 0.5 mM

[c] The apparent electron utilization efficiency is calculated by the ratio of the given electron equivalent of pollutant mineralization/obtained electron equivalent of oxidant decomposition. In the HEAs–PMS system, the apparent electron utilization efficiency is calculated as (1.067/0.5) × 100% = 213.4%. A similar phenomenon of high apparent electron utilization efficiency has also been reported in previous studies in heterogeneous systems.

Supplementary Table 7. The concentration of metal ion in the outlet streams solution of continuous-flow reactor system. Dosage: $[Phenol]_0$: 0.1 mM, PMS: 0.25 mM L^{-1}, catalyst: 150 mg.

Bed volume	Cu (mg L^{-1})	Pd (mg L^{-1})	Fe(mg L^{-1})	Co(mg L^{-1})	Ni(mg L^{-1})
454.75	0.013	0.001	0.042	0.005	0.005
1364.26	0.007	0.001	0.019	0.003	0.004
2273.76	0.006	0.001	0.012	0.003	0.003
3183.27	0.003	0.001	0.005	0.002	0.002
4547.52	0.001	0.002	0.004	0.001	0.001
6821.28	0.001	0.003	0	0	0
9095.04	0	0.004	0	0	0
11368.8	0	0.004	0	0	0
13642.56	-0.001	0.004	0	0	0
15916.33	0	0.005	0	0	0
18190.09	-0.001	0.005	0	0	0
20463.85	0	0.005	0	0	0
22737.61	-0.001	0.005	0	0	0
25011.37	-0.001	0.005	0	0	0
27285.13	-0.001	0.006	0	0	0
29558.89	-0.001	0.006	0	0	0
31832.65	0	0.006	0	0.001	0.001
34106.41	0	0.006	0	0.001	0
36380.17	-0.001	0.006	-0.003	0	0

Supplementary References

1. Chen, Z. *et al.* Single-atom Mo–Co catalyst with low biotoxicity for sustainable degradation of high-ionization-potential organic pollutants. *Proc. Natl. Acad. Sci. U.S.A.* **120**, e2305933120 (2023).
2. Wang, Z. *et al.* Cobalt Single Atoms Anchored on Oxygen-Doped Tubular Carbon Nitride for Efficient Peroxymonosulfate Activation: Simultaneous Coordination Structure and Morphology Modulation. *Angew. Chem. Int. Ed.* **61**, e202202338 (2022).
3. Yang, P., Long, Y., Huang, W. & Liu, D. Single-atom copper embedded in two-dimensional MXene toward peroxymonosulfate activation to generate singlet oxygen with nearly 100% selectivity for enhanced Fenton-like reactions. *Appl. Catal. B Environ.* **324**, 122245 (2023).
4. Liang, X. *et al.* Coordination Number Dependent Catalytic Activity of Single-Atom Cobalt Catalysts for Fenton-Like Reaction. *Adv. Funct. Mater.* **32**, 2203001 (2022).
5. Zhao, Z. *et al.* Construction of dual active sites on diatomic metal (FeCo−N/C-x) catalysts for enhanced Fenton-like catalysis. *Appl. Catal. B Environ.* **309**, 121256 (2022).
6. Zhang, D. *et al.* MOFs-derived magnetic C@Cu–Ni bimetal particles: An efficient peroxymonosulfate activator for 2,4,6-trichlorophenol degradation. *Chemosphere* **269**, 129394 (2021).
7. Yang, J. *et al.* Insights into the role of dual reaction sites for single Ni atom Fenton-like catalyst towards degradation of various organic contaminants. *J. Hazard. Mater.* **430**, 128463 (2022).
8. Feng, Y., Lee, P.-H., Wu, D. & Shih, K. Surface-bound sulfate radical-dominated degradation of 1,4-dioxane by alumina-supported palladium (Pd/Al_2O_3) catalyzed peroxymonosulfate. *Water Res.* **120**, 12–21 (2017).
9. Zhou, X. *et al.* Catalytic hydrodechlorination and advanced oxidation processes of 2,4-dichlorophenoxyacetic acid over CMK-3 supported catalyst: The bi-functional effect of metal Pd. *Chem. Eng. J.* **402**, 126175 (2020).
